# Endotoxin retention in adipose tissue leading to persistent hypotension in catecholamine-dependent acute obstructive suppurative cholangitis

**DOI:** 10.1515/jtim-2025-0053

**Published:** 2025-12-22

**Authors:** Kun Li, Sibo Zhu, Chun Ye, Yue Ding, Jiangxi Liu, Yufeng Wang, Zhiyong Wang, Wenfeng Li, DongHua Yang, Xiuyan Wang, Rui Lin, Baomin Shi

**Affiliations:** General Surgery Department, Tongji Hospital, School of Medicine, Tongji University, Shanghai, China; Department of General Surgery, Tongren Hospital, School of Medicine, Shanghai Jiao Tong University, Shanghai, China; MOE Key Laboratory of Contemporary Anthropology, School of Life Sciences, Fudan University, Shanghai, China; Surgery Department, Chongyi County People's Hospital, Ganzhou, Jiangxi Province, China; Administrative Dean, New York College of Traditional Chinese Medicine, New York, NY, USA; Ultrasound Department, Tongji Hospital, School of Medicine, Tongji University, Shanghai, China; Department of General Surgery, Xinhua Hospital, School of Medicine, Shanghai Jiao Tong University, Shanghai, China

**Keywords:** adipose tissue, endotoxin reservoir, catecholamine-dependent acute obstructive suppurative cholangitis, long-term hypotension, interleukin-1β

## Abstract

**Background and objectives:**

Catecholamine-dependent acute obstructive suppurative cholangitis (CD-AOSC) is a severe form of cholangitis characterized by refractory hypotension. This study aimed to investigate its underlying pathogenic mechanisms.

**Methods:**

We conducted a retrospective case-control study involving 345 patients with biliary infections treated at Tongji Hospital, Tongji University, between 2012 and 2022. Patients were divided into three groups: chronic cholecystitis (CC; negative control), acute obstructive suppurative cholangitis (AOSC; positive control), and CD-AOSC (case group). Clinical and laboratory data were analyzed. Endotoxin levels in subcutaneous adipose tissue were measured, and transcriptomic analysis was performed to explore potential mechanisms. A rat model of cholangitis was used to validate findings related to hypotension in CD-AOSC.

**Results:**

Patients with CD-AOSC exhibited slower endotoxin clearance from adipose tissue (1^st^ week *vs*. 2^nd^ week: 72.7 ± 4.0 EU/g *vs*. 23.3 ± 3.2 EU/g) and milder inflammation (Immune cell count: 665.4 ± 179.1, Immunodeconv analysis: 0.3 ± 0.1) compared to AOSC patients (1^st^ week *vs*. 2^nd^ week: 9.7 ± 2.6 EU/g *vs*. 2.6 ± 0.7 EU/g, Immune cell count: 1344.0 ± 599.3, Immunodeconv analysis: 0.6 ± 0.1). This slower clearance of endotoxin allowed adipose tissue to function as an endotoxin reservoir. Persistent endotoxin release resulted in prolonged activation of the interleukin-18 (IL-18)/IL-18 receptor accesary protein (IL-18RAP) pathway, thereby sustaining inflammation and contributing to extended hypotension. The rat model confirmed the role of the interleukin-1β (IL-1β) inhibitor in decelerating endotoxin release and maintaining activation of the IL-18/IL-18RAP/interferon-γ (IFN-γ) pathway.

**Conclusions:**

Persistent hypotension in CD-AOSC appears to result from sustained activation of the IL-18/IL-18RAP pathway, driven by prolonged endotoxin release from adipose tissue. These findings highlight the potential of "Hours Exchanged Against (severity) Loss (HEAL)" strategy, in which early modulation of endotoxin release from adipose tissue by IL-1β inhibition during the initial phase of sepsis in cholangitis may improve patient survival.

## Introduction

Acute obstructive suppurative cholangitis (AOSC) is primarily caused by biliary calculi and is a significant etiologic factor for sepsis. AOSC patients with septic shock always exhibit Charcot’s triad, characterized by abdominal pain, jaundice, and fever. The urgency of this condition cannot be overstated, as without immediate decompression of the biliary tract, patients are at risk of progressive multi-organ dysfunction, including renal and respiratory failure. The associated mortality rate is alarmingly high, ranging from 13% to 88%.^[1]^ In our clinical observation, the blood pressure of the patients usually recovered from shock within approximately four days following biliary decompression surgery. However, we noted some AOSC patients have persistent hypotension for several weeks after surgery and cannot be weaned off catecholamine pump despite successful biliary drainage. Approximately one week after biliary decompression, these patients demonstrate near-normal cognitive function, body temperature, and most of biochemical parameters, except for blood pressure. This subset of AOSC patients requires continued low-dose catecholamine infusion for a prolonged period, during which they exhibit mild abnormality in blood inflammatory markers but normal appetite and bowel function. Daily bile output *via* T-tube drainage averages 200 mL. Abrupt discontinuation of the catecholamine pump precipitates a rapid relapse into shock. Remarkably, these patients stabilize spontaneously several weeks later and no longer require catecholamine support.

We conducted a comprehensive literature search using the terms “long-term hypotension”, “prolonged hypotension”, “long-term shock”, and “prolonged shock” across databases including PubMed, Cochrane, Embase, and Web of Science to identify reports of this uncommon manifestation of AOSC. Despite reviewing over 20,000 articles, we found no directly relevant literature to the phenomenon we described. Consequently, we propose the term “catecholamine dependent AOSC (CD-AOSC)” to describe this distinct clinical phenomenon. In the past 10 years, there were about 28,000 AOSC patients in Tongji Hospital and in which five were diagnosed as CD-AOSC.

CD-AOSC can be defined as a condition observed in patients with acute cholangitis who, despite symptomatic relief and improved blood biochemical indicators following biliary drainage surgery, continue to require catecholamine vasopressors to maintain blood pressure for an extended period. This dependence typically lasts for about a month, during which interruption of catecholamine infusion leads to immediate hypotension and syncope. After this period, patients suddenly recover and no longer need catecholamines.

To elucidate the underlying mechanisms of CD-AOSC, we conducted a single-center, retrospective case-control study.

## Materials and methods

### Experimental design

**Figure 1:**
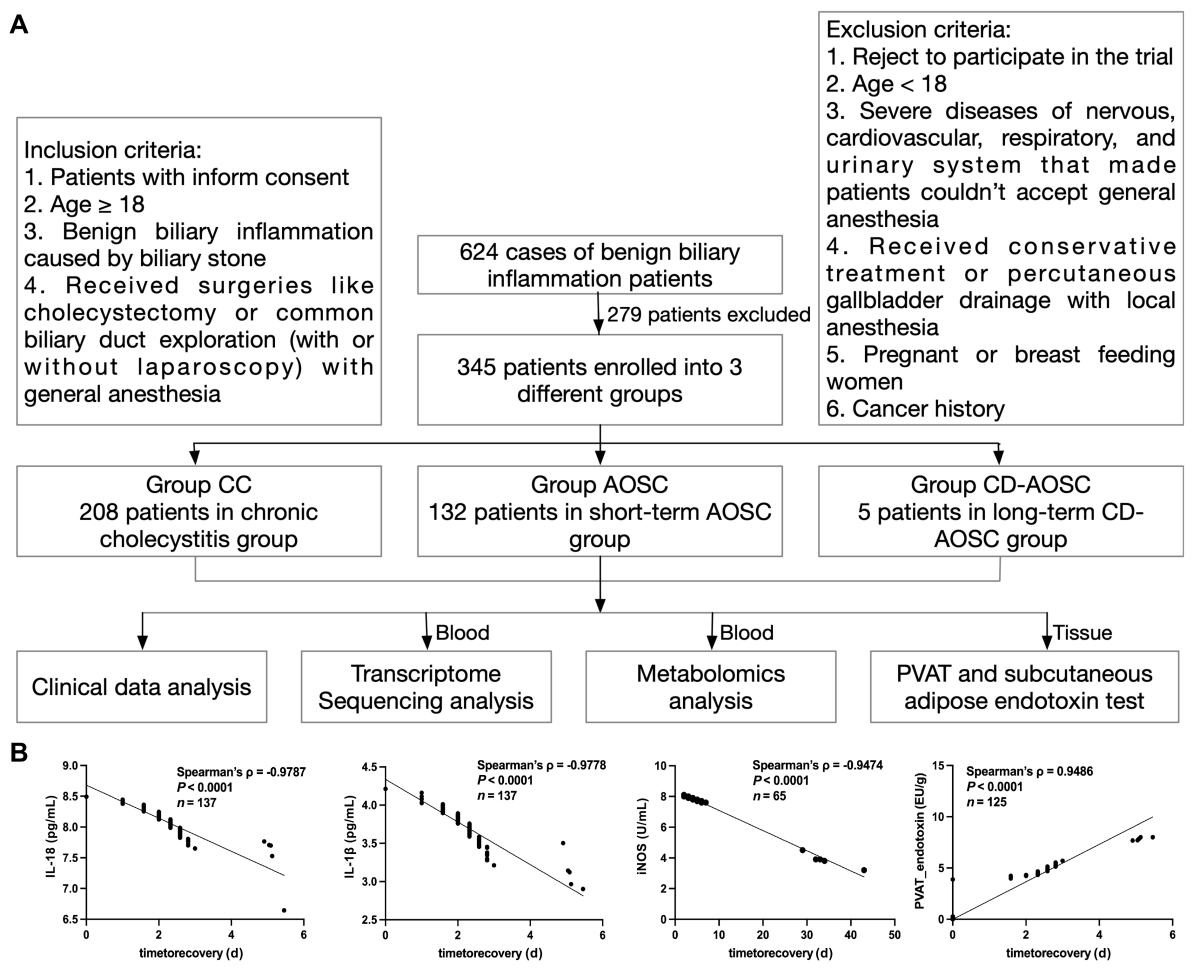
Experimental design and Pearson Correlation Analysis of clinical variables and Blood Pressure Recovery Time. (A). Inclusion and exclusion criteria for patients in different groups. (B). PVAT endotoxin and endotoxin in adipose showed strong positive correlation with time for recovery. IL-18, IFN-γ, and iNOS showed strong negative correlation with time for recovery (Spearman ρ represent Spearman correlation coefficient). CC: chronic cholecystitis; AOSC: acute obstructive suppurative cholangitis; CD-AOSC: catecholamine-dependent acute obstructive suppurative cholangitis; IL-18: interleukin-18; IFN-γ: interferon-γ; iNOS: inducible nitric oxide synthase.

To elucidate the underlying mechanisms of CD-AOSC, we conducted a retrospective cohort study dividing patients into 3 groups, *i.e*. patients with chronic cholecystitis (Group chronic cholecystitis [CC]), those with septic shock conforming to Grade II/III of the Tokyo Guidelines for AOSC (Group AOSC),^[2]^ and those with our newly defined CD-AOSC. In our retrospective cohort, we had 232 individuals with chronic cholecystitis and 387 patients with Grade II/ III AOSC, which included 5 CD-AOSC from January 1, 2012 to December 31, 2022 in Tongji Hospital affiliated to Tongji University. The specific inclusion and exclusion criteria were depicted in the flowchart (Figure 1A). All patients in the CD-AOSC and AOSC groups underwent emergency common bile duct exploration surgery (CBDE) and laparoscopic CBDE (LCBDE) within 24 h of admission. Finally, the study comprised three distinct groups with 208 chronic cholecystitis patients undergoing laparoscopic cholecystectomy in Group CC, 132 Grade II/III AOSC patients who required short-term catecholamine support (average 3.5 ± 1.4 days) post-biliary drainage surgery in Group AOSC, and 5 patients necessitating prolonged catecholamine-dependent management for septic shock (averaging 34 ± 5.3 days) following biliary drainage surgery in Group CD-AOSC. Blood and adipose tissue samples from all patients were cryopreserved and stored in a biobank for subsequent investigations.

**Figure 2:**
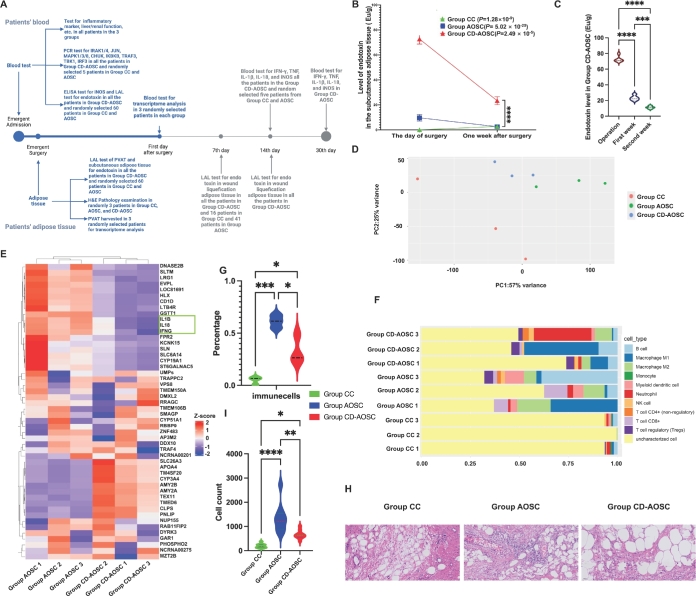
Reduced inflammation severity slows endotoxin elimination in adipose tissue of the CD-AOSC group. (A) Flowchart of the patient study design. (B) Endotoxin levels in subcutaneous adipose tissue on surgery day and one week post-surgery in patients with wound liquefaction. Endotoxin levels were significantly different between Group CD-AOSC and Group AOSC, as well as between Group CD-AOSC and Group CC after one week. (C) Two-week endotoxin level change in the CD-AOSC group, showing a slow but significant decrease post-surgery. (D) PCA clustering of PVAT transcriptome sequencing revealed significant differences among the three groups. (E) Heatmap of the top 25 up- and downregulated DEGs in PVAT between Group CD-AOSC and AOSC (*n* = 3 per group). IL-1β, IL-18, and IFN-γ (green box) were the most downregulated genes in Group CD-AOSC. (F) Immunedeconv analysis of PVAT transcriptome data showed higher immune cell infiltration in Group AOSC compared to Group CD-AOSC and CC. (G) Statistical analysis of immune cell proportions from Figure 2F. (H) Representative H&E staining images of PVAT in Groups CC, AOSC, and CD-AOSC (X200). (I) Quantification of immune cell counts from H&E images (Figure S4-6) showed significantly lower infiltration in Group CD-AOSC than AOSC but higher than CC (^*^P < 0.01, ^**^P < 0.001, ^***^P < 0.0001, ^****^P < 0.0001). CC: chronic cholecystitis; AOSC: acute obstructive suppurative cholangitis; CD-AOSC: catecholamine-dependent acute obstructive suppurative cholangitis; PCR: polymerase chain reaction; CHUK: component of inhibitor of nuclear factor kappa B kinase complex; IKBKB: inhibitor of nuclear factor kappa B kinase subunit beta; TRAF3: tumor necrosis factor receptor associated factor 3; TBK1: TRAF family member associated nuclear factor kappa B activator binding kinase 1; IRF3: interferon regulatory factor 3; ELISA: enzyme-linked immunosorbent assay; iNOS: inducible nitric oxide synthase; LAL: limulus amoebocyte lysate; PVAT: perivascular adipose tissue; IFN-γ: interferon-γ; TNF: tumor necrosis factor; IL-1β: interleukin-1β.

All research was conducted in accordance with both the *Declarations of Helsinki and Istanbul*. All participants underwent general anesthesia and provided informed consent. The Tongji University Tongji Hospital Ethics Review Committee granted approval for this study (K-W-2023-019). The experimental design workflow for both clinical and animal studies were shown in Figures 2A and 5A.

### Sample and clinical data collection, and statistical analysis

Upon emergent admission, comprehensive blood tests were performed on all patients to assess a range of parameters, including inflammation markers, liver and renal function, hormonal levels, and endotoxin levels. These tests included measurements of hemoglobin (Hb), platelets (PLT), hematocrit (HCT), alanine aminotransferase (ALT), aspartate aminotransferase (AST), blood urea nitrogen (BUN), creatinine level, C-reactive protein (CRP), serum amyloid A (SAA), heparin-binding protein (HBP), total bilirubin and direct bilirubin (TBIL and DBIL), normetanephrine, metanephrine, adrenocorticotropic hormone (ACTH), thyroid-stimulating hormone (TSH), angiotensin I/II, aldosterone, cortisol, brain natriuretic peptide (BNP), interferon-α (IFN-α), interferon-γ (IFN-γ), tumor necrosis factor (TNF), and a spectrum of interleukins (IL), such as IL-1β, IL-2, IL-4, IL-5, IL-6, IL-8, IL-10, IL-12, IL-17. We also assessed the messenger ribonucleic acid (mRNA) levels of interleukin 1 receptor associated kinase 1 (IRAK1), IRAK4, Jun proto-oncogene (JUN), component of inhibitor of nuclear factor kappa B (NF-κB) kinase complex (CHUK), inhibitor of NF-κB kinase subunit beta (IKBKB), tumor necrosis factor receptor associated factor 3 (TRAF3), TRAF family member associated nuclear factor kappa B activator binding kinase 1 (TBK1), and interferon regulatory factor 3 (IRF3) in blood samples from all patients in Group CD-AOSC and random five patients in Group CC and AOSC prior to biliary drainage surgery.

An additional 3 mL of blood was drawn from each patient on the day of emergent admission before surgery. After centrifugation, the plasma was stored at -80 ℃ for enzyme-linked immunosorbent assay (ELISA) testing to quantify inducible nitric oxide synthase (iNOS) level in all 5 patients in Group CD-AOSC and randomly selected 60 patients in the other two groups from our sample repository. Concurrently, we meticulously collected 1 cm samples of perivascular adipose tissue (PVAT) from around the gallbladder artery and subcutaneous adipose tissue from incision sites during surgery.^[3]^ These samples were immediately cryopreserved in -80 °C for future limulus amoebocyte lysate (LAL) endotoxin testing in all patients in Group CD-AOSC and randomly selected 60 patients in the other two groups from our sample repository. The PVAT were used for transcriptome analysis in randomly selected 3 patients from three groups.

The blood collected from patients on the first day after biliary drainage surgery is likely to retain the most critical pathways involved in CD-AOSC. To ensure uniformity, 10 mL of blood drawn on the first day post-surgery from three randomly selected patients within each of the three groups were retrieved from the biobank. These samples were then centrifuged, and the separated plasma and buffy coat were stored at -80 °C for future analysis. Subsequently, detailed transcriptome analyses were performed on these frozen samples. This approach provided comprehensive insights into the molecular profiles associated with CD-AOSC.

In clinical observations, fat liquefaction at the incision site was noted in all Group CD-AOSC patients, 16 patients in Group CC, and 41 patients in Group AOSC within one week post-surgery. Accordingly, we collected subcutaneous fat tissue from the incision sites of patients who experienced fat liquefaction first and second week post-operatively before wound healing and subjected these samples to LAL testing to assess endotoxin levels.

Fourteen days and thirty days after surgery, the blood samples were collected from all the patients in the Group CD-AOSC and randomly selected five patients from Group CC and AOSC. IFN-γ, TNF, IL-1β, IL-18, and iNOS were tested using these blood samples.

In univariable analysis among the three groups, the categorical data were analyzed by Pearson’s Chi-squared test with Yates’ continuity correction, and continuous data, of which normality was not assumed, were by Kruskal-Wallis rank sum test in one way analysis of variance. The *P* value smaller or equal to 0.05 was the criteria for statistical significance. We have applied the Benjamini-Hochberg method to control the false discovery rate and updated the manuscript accordingly.

Since the biomarker levels in the CD-AOSC group were generally intermediate between those in the negative control group (Group CC) and the positive control group (Group AOSC), we aimed to improve the prediction of time to recovery in CD-AOSC patients. To this end, we first identified variables that showed significant differences among the three groups in the univariate analysis. These variables were then subjected to pairwise comparisons between Group CD-AOSC and Group AOSC. Variables that remained significantly different were subsequently included in the construction of the multivariable linear regression model. A stepwise selection method based on the Akaike Information Criterion (AIC) was employed to determine the most parsimonious model, using backward selection strategy *via* the stepAIC () function from the MASS package (version 7.3-60.2).

After model selection, we assessed multicollinearity among the final predictors by calculating the variance inflation factor (VIF) using the vif () function from the car package. A VIF greater than 5 was considered indicative of problematic multicollinearity.

Model diagnostics were performed to evaluate assumptions of linearity, normality, and homoscedasticity of residuals. Standard residual plots, Q-Q plots, scale-location plots, and Cook’s distance were examined to detect potential outliers or influential observations. All analyses and visualizations were conducted in R software, and statistical significance was defined as *P* < 0.05.

To address potential multicollinearity and assess the robustness of variable selection, we performed a regularization-based analysis using Least Absolute Shrinkage and Selection Operator (LASSO) regression. The LASSO model was fitted using the glmnet package in R (version 4.1), with 10-fold cross-validation to determine the optimal penalty parameter (λ). The model was constructed using the same set of candidate predictors as in the linear regression, with the outcome variable being time to blood pressure recovery. Variables with non-zero coefficients under the optimal λ were considered robust predictors.

### Random forests analysis and spearman correlation analysis

Random forests (RF) are a non-parametric statistical method requiring no distributional assumptions on covariate relation to the response. A Random Forest was grown by bagging (L. Breiman 1996a) a collection of classification and regression trees (CART). The ggRandomForests package is the tool we used for visually growing random forest models. It’s structured to extract intermediate data objects from randomForestSRC objects and generates figures using the ggplot2 graphics package.

To assess the stability and uncertainty of importance rankings, 100 bootstrap replicates were performed. In each iteration, the model was refit on a bootstrap sample, and permutation importance was computed. The mean and standard error (SE) of each variable’s variable importance (VIMP) across replications were used to derive 95% confidence intervals. The top 10 variables were visualized using bar plots with error bars representing ±1.96 × SE.

To assess model generalizability and address the risk of overfitting, we performed 10-fold cross-validation using the caret package in R. The model was trained on 137 samples with 13 predictors. Model performance was evaluated across different values of the mtry tuning parameter. As mtry increased from 2 to 13, the root mean square error (RMSE) progressively decreased and the R^2^ increased, indicating improved model accuracy. Specifically, the model achieved the lowest RMSE (1.090), highest R^2^ (0.985), and lowest mean absolute error (MAE = 0.518) when mtry was set to 13. Therefore, the optimal model was selected based on the minimum RMSE criterion with mtry = 13.

The data of variables identified as top blood pressure recovery predictors in both stepwise multivariable regression model and the random forest model were imported into GraphPad Prism 9.5, with the X-axis representing “time-to-recovery” and the Y-axis representing the study variable. A scatter plot was generated, and Spearman correlation analysis was performed. The correlation coefficient (Spearman ρ value) and significance level (*P*-value) were directly labeled on the chart.

### RNA extraction and library preparation for transcriptome sequencing

RNA integrity and total amounts were evaluated using the Bioanalyzer 2100 system’s RNA Nano 6000 Assay Kit (Agilent Technologies). Total RNA was used to purify mRNA using poly-T oligo-attached magnetic beads, followed by fragmentation in First Strand Synthesis Reaction Buffer under high temperature. First strand complementary deoxyribonucleic acid (cDNA) synthesis involved a random hexamer primer and moloney murine leukemia virus (M-MuLV) Reverse Transcriptase, with subsequent RNA degradation by ribonuclease H (RNaseH). Second strand cDNA was synthesized using DNA Polymerase I and deoxy-ribonucleoside triphosphate (dNTP), and overhangs were blunted *via* exonuclease/polymerase. Post-adenylation of DNA fragment ends, an adaptor with a hairpin loop structure was ligated for hybridization. cDNA fragments (370~420 bp) were selected and purified using the AMPure XP system (Beckman Coulter, Beverly, USA), followed by polymerase chain reaction (PCR) amplification. The PCR products were purified, and the resultant library was sequenced on the Illumina NovaSeq 6000.

### mRNA Transcriptome Data Analysis

High-throughput sequencer image data were converted to sequence reads using CASAVA base recognition. Raw fastq format data were processed with fastp software, removing adapters, *N* base reads, and low-quality reads to yield clean data, alongside Q20, Q30, and glucocorticoids (GC) content calculations. Downstream analyses utilized this high-quality clean data.

The reference genome index was built using Hisat2 (v2.0.5), with paired-end clean reads aligned to the reference genome using the same tool. Read numbers mapped to each gene were counted using FeatureCounts (v1.5.0-p3), and gene transcripts per million (TPMs) were calculated. Differential expression analysis between two conditions/ groups (two biological replicates each) was conducted using the DESeq2 R package (1.38.3), adjusting P-values with the Benjamini-Hochberg method. Significant differential expression was identified with Padj ≤ 0.05 and|log2 (foldchange)|≥ 1.^[2,3]^ Heatmaps were generated using R packages: ComplexHeatmap (2.14.0), pheatmap (1.0.12), and ggplot2 (3.4.2). Immunedeconv (2.1.0) and gene set variation analysis (GSVA) (1.46.0) assessed immune cell enrichment levels and correlations with targeted genes. Gene ontology (GO) and kyoto encyclopedia of genes and genomes (KEGG) enrichment analyses were conducted using ClusterProfiler (4.7.1.003) and DOSE (3.24.2). Gene set enrichment analysis (GSEA) software (4.3.2) enriched pathways (C7. immunesigdb) and related genes in transcriptome data.

### Modeling of biliary tract infection in Rats

The experimental protocol was approved by the Ethical Committee of Tongji Hospital of Tongji University for Animal Care, Shanghai, China. Seventy male Sprague-Dawley rats (SD, body weight 280-320 g), had free access to food and tap water until the day of experiment. Seventy male Sprague-Dawley rats were randomly divided into four groups: Group A: Open-close group (*n* = 10), Group B: CBD ligation group (*n* = 20), Group C: CBD ligation plus lipopolysaccharides (LPS) injection into the common bile duct in the operation group (*n* = 20), Group D: CBD ligation plus LPS injection into the common bile duct in the operation and IL-1β inhibitor intraperitoneal injection twice a week group (*n* = 20). Blood pressure was monitored preoperatively and daily from postoperative day 1 to day 7 using a non-invasive tail-cuff system (Yuyan Instruments Co., Shanghai, China). Before each measurement, rats were warmed at 37 °C for 10 min to enhance tail vasodilation and reduce variability. To minimize stress-induced fluctuations, animals were acclimated to the restraining device for 30 min prior to recording. At each time point, the final blood pressure value was calculated as the average of at least three stable readings per rat.

Animals were anesthetized with 10% chloral hydrate (Hubio, Shanghai, China) at 5 g/ kg ip. After anesthesia, Group A, B, C, and D were established under sterile conditions according to the following methods (Figure 5A). In the Group A, the abdominal cavity was excised along the right inferior margin of liver, the tissues around the common bile duct were dissected bluntly, and then, the abdominal cavity was closed. In the Group B, C, and D, the abdominal cavity was excised along the right inferior margin of liver. Then, the common bile duct was ligated with double lines. After tying the knots, 0.2 mL 2 mg/mL LPS and 0.2 mL air were injected into the common bile duct in the Group C and D, while 0.2 mL 0.9% sodium chloride was injected into the common bile duct in the Group B. The InVivoMab anti-mouse/rat IL-1β (Bio X Cell, United States) was administered intraperitoneally at a dosage of 1 mg/ kg and twice a week (day 1 and 5) to the rats in Group D. In contrast, Group A, B, and C received an equivalent volume of 0.9% sodium chloride intraperitoneally twice a week (day 1 and 5) prior to sacrifice. All the rats survived were sacrificed on day 7. The LPS was extracted from Escherichia coli serotype O55: B5 (Ita Biotechnology Co., Ltd, Beijing, China).

### Rat sample collection and analysis timeline for PVAT, blood, and liver tissues (Figure 5A)

PVAT was harvested from rats on day 0 prior to surgery, as well as on days 1 and 7 post-surgery, and subjected to the LAL test. Blood samples were collected on day 7 post-euthanasia for quantitative PCR (qPCR) and ELISA analyses. Additionally, three liver tissues from each group were harvested on day 7 for hematoxylin and eosin (H & E) staining.

For all measured variables across Group A, B, C, and D, normality was assessed using the Shapiro-Wilk test. All variables demonstrated approximate normal distributions, with *P*-values < 0.05. Therefore, comparisons of continuous variables among groups were performed using one-way analysis of variance (ANOVA). For post hoc pairwise comparisons and adjustment for multiple testing, standard correction methods provided in GraphPad Prism (such as Tukey’s adjustment) were applied.

### Reverse transcription‑quantitative polymerase chain reaction (RT‑qPCR)

Total RNA was isolated from blood samples from all patients in Group CD-AOSC and randomly selected 5 patients in Group CC and AOSC using TRIzol (Invitrogen; Thermo Fisher Scientific, Inc.) and reverse transcribed into cDNA using a PrimeScript™ RT reagent Kit (cat. no. RR047A; Takara Bio, Inc.), according to the manufacturer’s protocols. Additionally, blood samples of all surviving rats in Group A, B, C, D were subjected to the same process of RNA extraction as that is patients. Briefly, RT was conducted at 37˚C for 15 min and 85 ˚C for 5 s, and the cDNA was stored at 4˚C until further use. qPCR was performed using SYBR^®^ Premix Ex Taq^™^ II (cat. no. RR820A; Takara Bio, Inc.) and an ABI 7500 Real‑time PCR system (Applied Biosystems; Thermo Fisher Scientific, Inc.) according to the manufacturer’s instructions. The primer sequences of human are as followed: IL1B 5’-AGATGAAGTGCTCCTTCCAGG-3’, 5 ’ -T G GT C G G AG AT T C G TAG C T G- 3 ’ , J U N 5 ’ -G A G A G C G G A C C T T A T G G C T A C- 3 ’ 5’-GTGAGGAGGTCCGAGTTCTTG-3’, CHUK 5 ’ - A T G A A G A A G T T G A A C C A T G C C A - 3 ’ , 5’-CCTCCAGAACAGTATTCCATTGC-3’, IKBKB 5 ’ -G G A A G T A C C T G A A C C A G T T T G A G- 3 ’ 5 ’ -G C AG G AC G AT G T T T T C T G G C T- 3 ’ , NF - κ B 1 5 ’ -G A AG C AC G A AT G AC A AG AG G C- 3 ’ 5 ’ -G C T T G G C G G A T T A G C T C T T T T- 3 ’ , I R A K 4 5 ’ -T G AT G G AG AT G AC C T C T G C T- 3 ’ 5 ’ -G G T G G A G T A C C A T C C A A G C A A - 3 ’ , I R A K 1 5 ′ -C A G T T C G C C G C C C T G A T- 3 ′ 5 ′ -T G C C A G G C T G T G A T G A T GT C- 3 ’ , TRAF3 5’-CTCACAAGTGCAGCGTCCAG-3’ 5 ’ -G C T C C A C T C C T T C A G C A G G T T- 3 ’ , T B K 1 5 ’ - AC G C AT G G G C AC AT C A AG A A - 3 ’ 5 ’-GT G C G T C A T A G C T T T T G T G G- 3 ’ , I R F 3 5 ’-TC T G C C C T C A A C C G C A A A G A A G- 3 ’ 5 ’ -T A C T G C C T C C A C C A T T G G T G T C- 3 ’ . The primer sequence of rat are as follows: IL1B 5 ’ -C A C C T C T C A A G C A G A G C A C A G- 3 ’ 5’-GGGTTCCATGGTGAAGTCAAC-3’, IL18 5’-TGCCTGATATCGACCGAACAGCCAAC-3’ 5’-ACAGATAGGGTCACAGCCAGTCCTCT-3’, interleukin 1 8 receptor accessory protein (IL18RAP) CCTATCTGATGTCCAGTGGT-5’, 3’-GGACAATGAATGGAGCCAGT; glyceraldehyde-3 -phosphate dehydrogenase ( G A P D H ) TGAATGACATCAAGAAGGTGGTGGAG-5’, IL18RAP 5’-CCTATCTGATGTCCAGTGGT-3’ 5 ’ -G G A C A A T G A A T G G A G C C A G T - 3 ’ , I F N- γ 5 ’-CC C T C T C T G G C T G T TAC T G C- 3 ’ 5 ’ -T T T C G T G T TAC C G T C C T T T T G- 3 ’ , i N O S 5 ’-CC T G G A G G T T C T A G A T G A G A G T - 3 ’ 5’-CTTCAGGTTATTGATCCAAGTG-3’, NFKB1 5 ’ -G C T G CC A A A G A A G G A C A C G A C A - 3 ’ 5 ’ -G G C A G G C T A T T G C T C A T C A C- A G- 3 ’ , 3’-TCCTTGGAGGCCATGTAGGCCAT. GAPDH was used as an endogenous control. The qPCR conditions comprised 95 ˚C for 30 s, and a total of 40 cycles at 95 ˚C for 5 s and extension at 60 ˚C for 34 s, followed by a dissociation curve analysis. All assays were performed three times. Relative expression levels were determined using the 2‑ΔΔCq method.

### Histological examination and quantification of inflammatory cells in PVAT and liver tissues

PVAT from randomly selected 3 patients in Group CC, AOSC, and CD-AOSC in the operations were subjected to H & E staining. The H & E images revealed variations in cell numbers among the groups, primarily due to differences in inflammatory cell counts. Three distinct 200X views of H & E staining from three different patients in each group were analyzed using ImageJ software. The Colour_Deconvolution2 extension of ImageJ was employed to quantify the number of cell nuclei in the pathological images (Supplementary Figure S3D). The average cell nucleus counts from the different groups were compared to reflect the varying severity of inflammation.

Rats that survived until day 7 in each group were euthanized. Liver tissue from randomly selected 3 rats in each group were collected and fixed in 10% neutral formalin. After 24 h of fixation, paraffin-embedded liver tissue blocks were prepared using conventional methods, sliced, stained with hematoxylin-eosin, and examined under a light microscope. To compare the severity of inflammation histologically, images of the portal area in X200 liver H&E stains from four different groups were captured using freeform snipping tools of Windows system. The average number of cell nuclei in the portal area, primarily consisting of inflammatory cells, was used to assess inflammation severity. Cell counts were performed using the same method as previously described (Supplementary Figure S3D) with ImageJ software.

### Enzyme-linked immunosorbent assay (ELISA)

On the day before operation, 2 mL blood was taken from all patients in Group CD-AOSC and randomly selected 60 patients in Group CC and AOSC, and centrifuged at 1000 ×*g* for 10 min at 4 °C. Serum was collected for subsequent detection for iNOS.

On day 7 postoperatively, 1.5 mL blood was taken from the rat tail vein and centrifuged at 1000 ×*g* for 10 min at 4 °C. Serum was collected for the subsequent detection for IL18, IL1B, IL18RAP, IFN-γ, iNOS and NF-κB1.

The inflammatory factors IL18, IL-1β, IL18RAP, IFN-γ, iNOS and NF-κB1 were detected using human or rat ELISA kit using SpectraMAX Plus384 (Molecular Devices [Shanghai] Co. Ltd). The levels of inflammatory factors were detected according to the instructions of ELISA kit (Chunmai Biotechnology Co., Ltd, Shanghai, China). Specifically, 50 μL of blank, standard, and sample solutions were added to the 48 well plate supplied in the reagent kit. Following 30 min of incubation at 37 °C, the 96 well plate was washed using buffer solution. With the exception of blank wells, each well was added with 100 μL of enzyme-labeled reagent, incubated, and then added with chromogenic agent. After the reaction was terminated, the absorbance was measured at 450 nm.

## Results

### PVAT endotoxin, IL-1β, IL-18, and iNOS are important markers for predicting the recovery time for blood pressure

The demographic data of the 345 patients from the three groups were shown in Table 1. There was no statistically significant difference among groups in gender, age, the history of high blood pressure, diabetes, and body mass index (BMI). However, there were significant differences among groups in systolic/diastolic blood pressure on admission, the surgery type, gallbladder gangrene discovered in operation, operation time, and time for blood pressure recovery after surgery.

**Table 1:** General information statistics for three groups

Item	CC (*n* = 208)	AOSC (*n* = 132)	CD-AOSC (*n* = 5)	FDR.adjusted.*P*
Gender				0.911
0	108 (51.9%)	67 (50.8%)	2 (40.0%)	
1	100 (48.1%)	65 (49.2%)	3 (60.0%)	
Gallbladder gangrene				0.001
0	208 (100.0%)	122 (92.4%)	4 (80.0%)	
1	0 (0.0%)	10 (7.6%)	1 (20.0%)	
High blood pressure				0.265
0	141 (67.8%)	101 (76.5%)	3 (60.0%)	
1	67 (32.2%)	31 (23.5%)	2 (40.0%)	
Diabetes				0.134
0	163 (78.4%)	107 (81.1%)	2 (40.0%)	
1	45 (21.6%)	25 (18.9%)	3 (60.0%)	
Surgery				0.001
0	0 (0.0%)	45 (34.1%)	2 (40.0%)	
1	0 (0.0%)	87 (65.9%)	3 (60.0%)	
2	208 (100.0%)	0 (0.0%)	0 (0.0%)	
Age	57.0 (51.0, 64.2)	58.0 (47.8, 66.0)	64.0 (56.0, 64.0)	0.911
Body Mass Index	23.2 (22.3, 24.0)	23.4 (22.0, 24.2)	23.7 (22.4, 24.2)	0.911
Time for BP Recovery	0.0 (0.0, 0.0)	3.5 (3.0, 4.0)	33.0 (32.0, 34.0)	0.001
Systolic Blood Pressure on Admission	108.5 (102.0, 116.2)	84.0 (83.0, 86.0)	85.0 (82.0, 87.0)	0.001
Diastolic Pressure on Admission	70.0 (66.0, 73.0)	40.0 (38.0, 42.0)	47.0 (45.0, 48.0)	0.001
Operation time (min)	61.0 (53.0, 70.0)	79.0 (71.0, 152.0)	78.0 (72.0, 149.0)	0.001

The information of group CC, AOSC, and CD-AOSC are illustrated in design of the experiment in method part. Body mass index (BMI) = Weight (kg)/Height^2^ (m^2^). The statistical numbers for numeric data shown in each cell of the table was in the form of Median (Interquartile Range). The numbers for category data were summarized in counts(percentage) form. Statistical method used for comparison of continuous or category data were listed in the method part. *P*-value ≤ 0.05 was statistically significant different. CC: chronic cholecystitis; AOSC: acute obstructive suppurative cholangitis; CD-AOSC: catecholamine-dependent acute obstructive suppurative cholangitis; HBP history: high blood pressure history; CBDE: common bile duct exploration; LCBDE: laparoscopic common bile duct exploration; LC: laparoscopic cholecystectomy; BP: blood pressure. The variables were coded as follows: in Gender, 0 represents female and 1 represents male; in Gallbladder gangrene, 0 indicates absence of gangrene and 1 indicates presence of gangrene; in High blood pressure and Diabetes, 0 indicates no history of hypertension and 1 indicates a history of hypertension; in Surgery, 0 indicates CBDE, 1 indicates LCBDE, and 2 indicates LC.

The statistics of laboratory tests among the three groups were shown in Table 2. There were statistically significant differences among groups in CRP,HBP, normetanephrine, metanephrine, cortisol, IFN-α, IFN-γ, IL-1β, IL-5, IL-6, IL-17, IL-18, iNOS, PVAT endotoxin, endotoxin in subcutaneous adipose, TBIL, DBIL, hemoglobin (Hb), ALT, AST, BUN, and creatinine (*P* ≤ 0.05).

**Table 2:** Statistics for laboratory tests among three groups

Item	CC (*n* = 208)	AOSC (*n* = 132)	CD-AOSC (*n* = 5)	FDR.adjust.*P*
CRP (mg/L)	3.5 (2.5, 4.5)	110.9 (89.4, 129.4)	91.9 (91.3, 98.4)	0.001
HBP (ng/mL)	7.0 (5.3, 8.7)	184.5 (169.5, 201.1)	129.1 (107.5, 130.3)	0.001
Normetanephrine (U/mL)	70.2 (64.9, 76.0)	133.4 (125.1, 141.1)	96.6 (94.7, 100.0)	0.001
Metanephrine (ng/mL)	1.1 (1.0, 1.2)	1.2 (1.0, 1.4)	0.8 (0.7, 1.1)	0.002
ACTH (pmol/L)	9.6 (7.8, 12.2)	10.2 (8.1, 12.0)	10.2 (6.3, 11.0)	0.721
TSH (mU/L)	6.7 (5.4, 7.7)	6.3 (5.5, 7.4)	6.2 (5.7, 7.3)	0.511
Angiotensin I (ng/L)	46.6 (33.8, 59.4)	51.9 (37.2, 62.5)	51.0 (45.9, 60.7)	0.143
Angiotensin II (ng/L)	38.2 (24.4, 49.9)	41.1 (31.2, 51.0)	54.2 (36.4, 57.2)	0.176
Aldosterone (pmol/L)	78.3 (57.3, 95.2)	74.5 (56.5, 87.5)	72.3 (51.2, 73.4)	0.363
Cortisol (U/L)	292.5 (192.8, 393.7)	495.1 (393.3, 572.7)	551.5 (377.9, 587.5)	0.001
BNP (pg/mL)	45.6 (34.7, 57.7)	41.2 (35.0, 52.4)	43.1 (42.2, 45.3)	0.162
IFN-α (kU/L)	4.1 (3.8, 4.5)	3.7 (3.4, 4.2)	3.9 (3.3, 4.0)	0.001
IFN-γ (U/L)	0.9 (0.8, 1.0)	13.1 (12.0, 13.8)	8.7 (8.1, 9.1)	0.001
TNF (μg/L)	2.2 (1.5, 2.7)	2.1 (1.6, 2.9)	2.6 (2.3, 3.7)	0.511
IL-1β (pg/mL)	0.2 (0.2, 0.2)	12.5 (11.0, 13.8)	7.7 (6.8, 7.8)	0.001
IL-2 (pg/mL)	3.5 (2.4, 4.4)	3.4 (2.5, 4.5)	3.5 (2.3, 3.7)	0.802
IL-4 (pg/mL)	4.3 (3.1, 5.3)	4.6 (3.7, 5.6)	5.1 (3.9, 5.3)	0.132
IL-5 (pg/mL)	1.4 (1.1, 1.8)	1.6 (1.3, 1.8)	1.2 (1.2, 1.3)	0.018
IL-6 (pg/mL)	9.4 (8.4, 10.5)	23.2 (17.8, 28.0)	14.1 (13.8, 16.9)	0.001
IL-8 (pg/mL)	10.4 (9.6, 11.4)	10.5 (8.5, 12.0)	11.6 (11.6, 12.0)	0.132
IL-10 (pg/mL)	6.6 (5.4, 7.5)	6.6 (5.9, 7.5)	6.2 (5.8, 6.5)	0.461
IL-12 (pg/mL)	1.8 (1.4, 2.1)	1.8 (1.6, 2.1)	2.1 (2.0, 2.1)	0.461
IL-17 (pg/mL)	11.7 (10.1, 13.6)	13.1 (11.8, 13.8)	12.7 (11.8, 12.8)	0.001
IL-18 (pg/mL)	27.9 (22.9, 35.3)	277.9 (253.6, 301.4)	206.9 (183.6, 208.1)	0.001
iNOS (U/mL)	0.2 (0.2, 0.2)	7.8 (7.8, 7.9)	3.9 (3.8, 3.9)	0.001
PVAT Endotoxin (EU/g)	0.1 (0.1, 0.2)	25.6 (19.4, 29.6)	233.3 (207.7, 256.1)	0.001
Endotoxin in Adipose (EU/g)	0.1 (0.1, 0.1)	7.7 (5.6, 10.1)	71.0 (70.6, 72.0)	0.001
Endotoxin tissue one in week subcutaneous after surgery adipose	1.9 (1.7, 1.9)	2.6 (2.3, 3.2)	22.7 (21.5, 24.3)	0.001
Endotoxin tissue two in week subcutaneous after surgery adipose	0.2 (0.1, 0.4)	0.2 (0.1, 0.3)	11.1 (10.5, 11.8)	0.001
Endotoxin in blood(EU/g)	0.2 (0.1, 0.3)	0.2 (0.1, 0.2)	0.2 (0.1, 0.3)	0.375
TBIL (μmol/L)	13.8 (12.9, 14.8)	136.8 (109.2, 160.8)	161.5 (107.3, 171.2)	0.001
DBIL (μmol/L)	4.9 (4.7, 5.1)	71.6 (64.9, 78.2)	87.2 (60.8, 92.2)	0.001
Platelet (109/L)	246.5 (219.9, 279.7)	252.7 (222.8, 279.0)	252.2 (246.1, 261.1)	0.721
Hb (g/L)	133.5 (129.0, 138.0)	131.0 (124.8, 135.0)	126.0 (126.0, 130.0)	0.001
Hematocrit (%)	42.8 (42.1, 43.8)	42.9 (41.8, 44.4)	41.5 (40.9, 45.2)	0.875
ALT (U/L)	33.0 (30.0, 36.0)	141.0 (126.0, 156.2)	176.0 (141.0, 178.0)	0.001
AST (U/L)	27.0 (24.0, 31.0)	116.0 (102.0, 132.2)	110.0 (109.0, 113.0)	0.001
BUN (mmol/L)	3.3 (2.7, 3.7)	7.2 (6.7, 7.6)	3.8 (3.1, 3.9)	0.001
Creatinine (μmol/L)	65.0 (58.5, 72.5)	103.0 (95.7, 108.8)	63.5 (61.7, 64.6)	0.001

The statistical numbers for numeric data shown in each cell of the table was in the form of Median(Interquantile Range). Statistical method used for comparison of continuous or category data were listed in the method part. *P*-value ≤ 0.05 was statistically significant different. CC: Chronic Cholangitis; AOSC: Acute Obstructive Suppurative Cholangitis; CD-AOSC: Catecholamine Dependent Acute Obstructive Suppurative Cholangitis; CRP: C-reactive protein; HBP: Heparin-binding protein; ACTH: Adrenocorticotropic hormone; TSH: Thyroid-stimulating hormone; BNP: B-Type natriuretic peptide; IFN: Interferon; TNF: Tumor necrosis factor; IL: Interleukin; iNOS: inducible Nitric oxide synthase; PVAT: Perivascular adipose tissue; TBIL: Total bilirubin; DBIL: Direct bilirubin; Hb: Hemoglobin; ALT: Alanine transaminase; AST: Aspartate aminotransferase; BUN: Blood urea nitrogen.

In Tables 1 and 2, Group CC served as the negative control group, aiming to eliminate potential confounding effects caused by anesthesia, surgery, and related factors. In contrast, Group AOSC functioned as a more informative positive control group. We observed that, for many markers of interest, the levels in the CD-AOSC group were intermediate between those in Group CC and Group AOSC. To facilitate the construction of predictive models, we extracted variables that exhibited statistically significant differences among the groups in Tables 1 and 2, and subsequently performed pairwise comparisons between Group AOSC and CD-AOSC. Variables showing significant differences in this comparison (Supplementary Table S1 and 2) were further incorporated into the development of future regression and Random Forest models.

The final stepwise multivariable regression model identified several significant predictors of time for blood pressure recovery. Notably, higher levels of PVAT_endotoxin (β = 0.036, *P* < 0.001), BUN (β = 1.657, *P* = 0.0044), creatinine (β = 0.096, *P* = 0.0029), and were positively associated with prolonged recovery. In contrast, IL-18 (β = −0.080, *P* < 0.001) and iNOS (β = −6.220, *P* < 0.001) showed strong and independent negative associations with recovery time. IL-1β (β = 0.338, *P* = 0.057) showed a marginal positive association, while IFN-γ (β = 0.461, *P* = 0.098) showed a marginal inverse association; neither reached statistical significance. Overall, the model exhibited excellent fit, with an adjusted R^2^ of 0.998 and an overall *P*-value < 2e-16 (Table 3).

**Table 3:** Stepwise multivariable regression model for recovery time prediction

Parameter	Estimate	Std. Error	*t* value	Pr (< |t|)
(Intercept)	54.05442	4.11591	13.13	< 2e-16^***^
IFN-γ (U/L)	-0.46090	0.27408	-1.68	0.09821 .
IL-1β (pg/mL)	0.33849	0.17452	1.94	0.05748 .
IL-18 (pg/mL)	-0.08014	0.00738	-10.86	2.1e-15^***^
iNOS (U/mL)	-6.21978	0.44204	-14.07	< 2e-16^***^
PVAT_endotoxin (EU/g)	0.03603	0.00965	3.74	0.00044^***^
BUN (mmol/L)	1.65656	0.55757	2.97	0.00437^**^
creatinine (μmol/L)	0.09585	0.03082	3.11	0.00294^**^

Signif. codes: 0 ‘***’ 0.001 ‘**’ 0.01 ‘*’ 0.05 ‘.’. Residual standard error: 0.338 On 56 degrees of freedom. Multiple R-squared: 0.998, Adjusted R-squared: 0.998. *F*-statistic: 5.32e+03 on 7 and 56 DF, *P*-value: < 2e-16. IL-1β: interleukin-1β; IFN-γ: interferon-γ; iNOS: inducible nitric oxide synthase; NF-κB: nuclear factor kappa-B; LPS: lipopolysaccharides; PVAT: Perivascular adipose tissue; BUN: blood urea nitrogen.

VIF analysis revealed severe multicollinearity among several predictors. Specifically, PVAT_endotoxin (VIF = 163.1), BUN (VIF = 168.2), iNOS (VIF = 126.7), creatinine (VIF = 82.9), IFN-γ (VIF = 71.0), IL-1β (VIF = 41.3), and IL-18 (VIF = 30.7) all exceeded conventional thresholds for multicollinearity. These findings indicate substantial intercorrelations among inflammatory and renal biomarkers, warranting cautious interpretation of individual effect sizes. (Supplementary Table S3)

Regression diagnostic plots (Supplementary Figure S1) supported the model’s assumptions. Residuals were approximately normally distributed (Q-Q plot) and evenly dispersed around the fitted line (Residuals *vs*. Fitted), with no clear pattern indicating heteroscedasticity (Scale-Location plot).

As a sensitivity analysis to address concerns regarding multicollinearity, we conducted LASSO regression with 10-fold cross-validation. The LASSO model retained a subset of predictors including IL-1β (β = 0.480), IL-18 (β = -0.044), iNOS (β = -6.523), and PVAT_endotoxin (β = 0.012), as well as IFN-γ, IL-5, IL-6, and HBP (Supplementary Table S4). Notably, variables with the highest VIFs in the linear model (*e.g*., BUN and creatinine) were eliminated by the LASSO penalty, suggesting they did not contribute significantly to the prediction of recovery time. The consistency of IL-1β, IL-18, iNOS, and PVAT_endotoxin as retained predictors in both the multivariable and LASSO models supports the robustness of their associations.

A random forest was grown for regression to pick out the explainable factors for the median time for blood pressure recovery using the independent predictor variables that had significant differences between Group CD-AOSC and AOSC listed in Supplementary Table S1 and S2. The final forest was built from 64 observations and 13 independent variables. It was constructed for the continuous variable of “time for blood pressure recovery” using 500 trees, randomly selecting 8 candidate variables at each node split, and terminating nodes with no fewer than 5 observations. The plot in Supplementary Figure S2A demonstrated that it didn’t take a large number of trees to stabilize the forest prediction error estimate.

The VIMP plot shown in Supplementary Figure S2B used bars to compare the scale of the error increase under permutation. The random forest model identified iNOS, BUN, endotoxin in subcutaneous adipose, PVAT endotoxin levels as the most important predictors of recovery time. As shown in Supplementary Figure S2B, the top 10 variables were ranked by their mean VIMP scores, with 95% confidence intervals derived from bootstrap-based standard errors. Variables such as creatinine, IFN-γ, normetanephrine, IL-1β, HBP, and IL-18 had relatively lower VIMP values and wider confidence intervals that overlapped zero, suggesting less stable importance in the model.

The minimal depth of variables was ranked from the most important at the top to the least at the bottom (Supplementary Figure S2C). The variables, including iNOS, BUN, IFN-γ, PVAT endotoxin, creatinine, endotoxin in subcutaneous adipose, IL-1β, and IL-18, on the left side of the dashed line indicated higher importance in the random forest.

The gg_minimal_vimp function was used to compare rankings between minimal depth and VIMP (Supplementary Figure S2D). Comparing Minimal Depth and Vimp rankings, points lower than the horizontal dashed line and left of the vertical dashed line, such as iNOS, BUN, IFN-γ, PVAT endotoxin, creatinine, Endotoxin in subcutaneous adipose, IL-1β, and IL-18, exhibited both low minimal depth and high VIMP, suggesting that they were not only frequently selected in early tree splits but also contributed significantly to model accuracy. These variables are thus considered the most predictive features in the dataset. The R-square of this random forest model was 0.87.

The factors identified by the stepwise multivariable regression, LASSO regression, and the random forest model were subsequently used in a Spearman correlation analysis to assess their relationship with blood pressure recovery time. PVAT endotoxin (Spearman ρ = 0.95) demonstrated a strong positive correlation with blood pressure recovery time. IL-1β (ρ = -0.98), IL-18 (ρ = -0.98), and iNOS (ρ = -0.95) demonstrated a strong negative correlation with blood pressure recovery time. (Figure 1B, *P* < 0.0001).

### Reduced severity of inflammation in adipose tissue may contribute to slower release of endotoxins in CD-AOSC

In Group CD-AOSC, all five patients experienced wound fat liquefaction approximately one week post-surgery, while 16 patients in Group CC and 41 in Group AOSC also suffered from this complication.

In these fat liquefaction patients, Group CC showed an increase in endotoxin levels, while Groups AOSC and CD-AOSC exhibited significant decreases in the subcutaneous adipose tissue between the time of operation and the first week after surgery (Figure 2B).

By the second week post-surgery, subcutaneous endotoxin levels in all fat liquefication patients of Group CC and AOSC were nearly normal, but remained significantly elevated in Group CD-AOSC, suggesting adipose tissue as an endotoxin reservoir in Group CD-AOSC patients (Figure 2C Table 4).

**Table 4:** The endotoxin level in subcutaneous adipose tissue

Endotoxin levels at different time points	CC (*N* = 16)	AOSC (*N* = 41)	CD-AOSC (*N* = 5)	*P* value
Endotoxin in subcutaneous fat tissue when surgery (EU/g)	0.110 (0.047)	7.739 (3.777)	72.740 (3.998)	< 0.001
Endotoxin in wound fat liquefication tissue first week after surgery (EU/g)	1.775 (0.242)	2.827 (0.646)	23.300 (3.196)	< 0.001
Endotoxin in wound fat tissue second week after surgery (EU/g)	0.263 (0.171)	0.237 (0.128)	11.220 (1.593)	< 0.001

The statistical numbers for numeric data shown in each cell of the table was in the form of mean(standard error). Statistical method used for comparison of continuous data were listed in the method part. *P* value ≤ 0.05 was statistically significant different. The endotoxin level in subcutaneous fat tissue of Group CD-AOSC were significantly higher than the other two groups at three different time points (*P*<0.001).

Principal component analysis (PCA) revealed distinct transcriptomic differences in PVAT samples across the three groups (Figure 2D). Among the top 25 differentially expressed genes between Group CD-AOSC and Group AOSC, IL-1Β, IL18, and IFN-γ were significantly lower in Group CD-AOSC (Figure 2E, green box). Compared to Group CC, Group CD-AOSC showed elevated levels of IL1B, IFN-γ, TNF, IL18RAP, IL6, and IL18 (Supplementary Figure S3C, green box). Immune cell deconvolution analysis of PVAT transcriptomes indicated a markedly higher proportion of immune cells in Group AOSC than in Group CD-AOSC and Group CC (Supplementary Table S5, Figure 2G, F).

H&E staining of PVAT samples revealed a heterogeneous mix of adipocytes, interstitial cells, and inflammatory cells in all groups (Figure 2H, S4-S6). Quantitative cell density analysis (Supplementary Figure S3D) demonstrated increased inflammation, as indicated by higher cell counts per field at 200× magnification. Group CD-AOSC had significantly higher cell density (665.4 ± 179.1 cells/field) than Group CC (201.4 ± 89.6 cells/field), but lower than Group AOSC (1344 ± 599.3 cells/field). These results suggest varying degrees of inflammation across the groups (Figure 2I).

Blood inflammatory markers exhibited comparable temporal trends across the three groups. As illustrated in Supplementary Figure S3A and S3B, the CD-AOSC group showed lower white blood cell (WBC) and CRP levels at admission and on postoperative day 3 compared to the AOSC group. However, in the CD-AOSC group, both markers (still higher than normal range) declined more gradually from day 3 to day 28, whereas levels in the AOSC group returned to baseline within the same period.

### IL1B and IL-18/IL18RAP pathway plays a significant role in leading to shock

Whole blood samples from the three groups were taken on the 1^st^ days after operation for transcriptome sequencing. The data of transcriptome were in good quality control (Supplementary Figure S3E, F).

**Figure 3:**
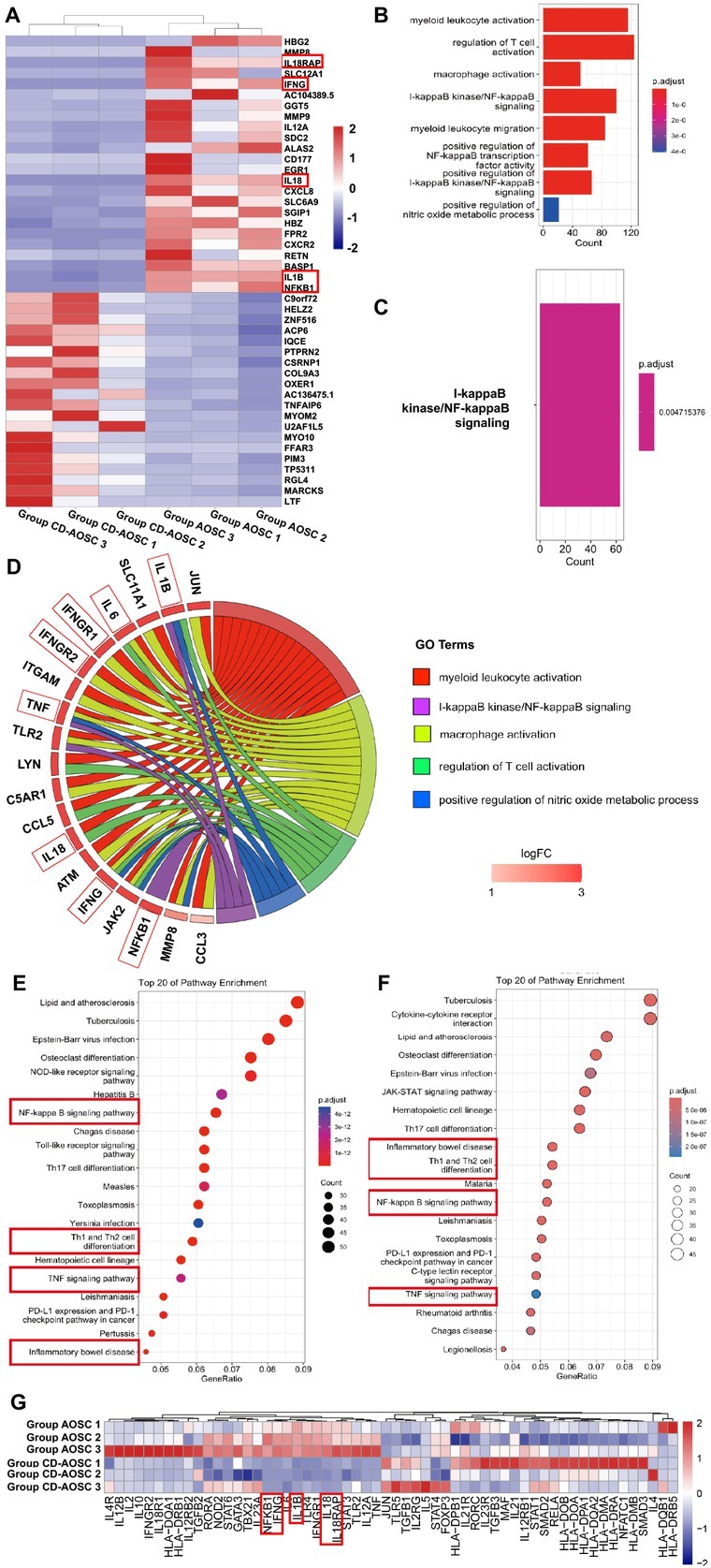
The “inflammatory bowel disease” KEGG pathway contains the most differentially expressed genes among the three groups. (A) Transcriptome analysis of patients’ blood showing the top 25 significantly up- and downregulated genes between Group AOSC and Group CD-AOSC. Genes in red boxes were significantly upregulated in Group AOSC. (B) Gene ontology analysis identified the top 8 enriched pathways in patients’ blood between Group CD-AOSC and Group CC. (C) The only significantly enriched pathway in gene ontology analysis between Group AOSC and Group CD-AOSC was the I-kappaB kinase/NF-kappaB signaling pathway. (D) Chord diagram illustrating key genes involved in the most relevant pathways. (E) KEGG pathway analysis of patients’ blood in Group CD-AOSC *vs*. Group CC identified the top 20 enriched pathways, including NF-kappaB signaling, Th1/Th2 cell differentiation, TNF signaling, and inflammatory bowel disease. (F) KEGG pathway analysis of Group AOSC vs. Group CD-AOSC identified the top 20 enriched pathways, notably inflammatory bowel disease, Th1/Th2 cell differentiation, NF-kappaB signaling, and TNF signaling. (G) Heatmap visualization of genes expression between Group AOSC and CD-AOSC related to the inflammatory bowel disease pathway (hsa05321), clustered by expression patterns. Th1 cell: type I helper cell; CC: chronic cholecystitis; AOSC: acute obstructive suppurative cholangitis; CD-AOSC: catecholamine-dependent acute obstructive suppurative cholangitis; CHUK: component of inhibitor of nuclear factor kappa B kinase complex; IKBKB: inhibitor of nuclear factor kappa B kinase subunit beta; TRAF3: tumor necrosis factor receptor associated factor 3; TBK1: TRAF family member associated nuclear factor kappa B activator binding kinase 1; IRF3: interferon regulatory factor 3; ELISA: enzyme-linked immunosorbent assay; iNOS: inducible nitric oxide synthase; LAL: limulus amoebocyte lysate; PVAT: perivascular adipose tissue; IFN-γ: interferon-γ; TNF: tumor necrosis factor; IL-1β: interleukin-1β; IL18RAP: interleukin 18 receptor accessory protein; KEGG: kyoto encyclopedia of genes and genomes.

The top 25 differentially expressed genes between Group CD-AOSC and AOSC are shown in Figure 3A. The differentially expressed genes between Group CD-AOSC and CC are shown in Supplementary Figure S7A.

IL18RAP, IFN-γ, IL18, IL1B, and NF-κB1 were significantly upregulated in Group AOSC compared with Group CD-AOSC (Figure 3A). IL1B, IFN-γ receptor 1 (IFN-γR1), IL18RAP, IFN-γ, IL18, and NF-κB1 were upregulated in Group CD-AOSC compared with CC (Supplementary Figure S7A).

These findings indicate that the IL1B/IL18/IL18RAP/ IFN-γ pathway was active after surgery.

### The enriched KEGG pathway of “inflammatory bowel disease” contained the most molecules found in differentially expressed genes among the three groups

In GO enrichment analysis of differentially expressed genes between Group CD-AOSC and CC, “myeloid leukocyte activation”, “regulation of T cell activation”, “macrophage activation”, “I-κB kinase/NF-κB signaling”, “myeloid leukocyte migration”, “positive regulation of NF-κB transcription factor activity”, “positive regulation of I-κB kinase/NF-κB signaling”, and “positive regulation of nitric oxide metabolic process” were the most interested pathways that enriched in Group CD-AOSC (Figure 3B, Supplementary Table S6). One and the same signaling pathway was also enriched in the differentially expressed genes between group CD-AOSC and AOSC (Figure 3C), namely “I-κB kinase/NF-κB signaling”(adjust *P* = 0.005), indicating NF-κB1 played an important role.

IL6, IFN-γR1/2, TNF, IL18, and IFN-γ were involved in myeloid leukocyte activation pathway. IL6, IFN-γR1/2, TNF, and IFN-γ were involved in macrophage activation. IL1B, IL6, IL18, and IFN-γ were involved in regulation of T cell activation pathway. IL1B, TNF, and IFN-γ were involved in positive regulation of nitric oxide metabolic process pathway. IL1B, TNF and NF-κB1 were involved in I-κB kinase/NF-κB signaling (Figure 3D).

The shared pathways enriched in KEGG between Group CD-AOSC and CC (Figure 3E) and between Group CD-AOSC and AOSC (Figure 3F) were “inflammatory bowel disease” pathway (Supplementary Figure S7B). This pathway contained the most differentially expressed genes found among groups (Figure 3A and Supplementary Figure S7). The heatmap of molecules participate in the “inflammatory bowel disease” revealed higher expression of NF-κB1, IFN-γ, IL18, IL1B, and IL18RAP in Group AOSC than in Group CD-AOSC (Figure 3G).

### Macrophages and Th1 cells play a crucial role in CD-AOSC

**Figure 4:**
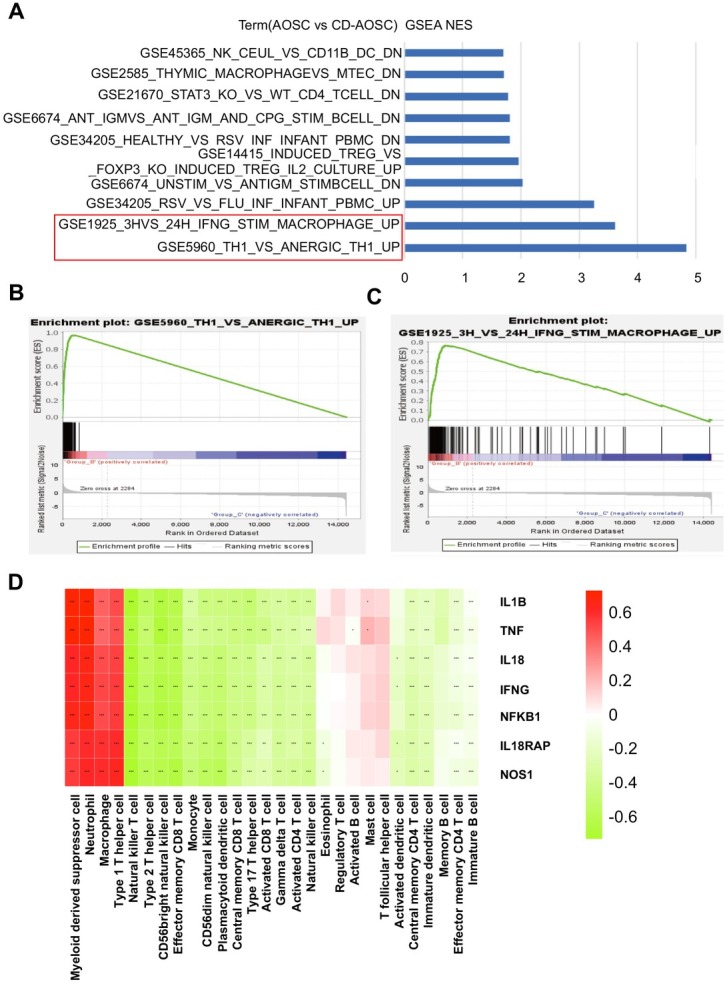
Macrophage and Th1 cells played a significant role in CD-AOSC. (A) GSEA analysis indicated significant upregulation of GSE1925 and GSE5960 in the blood transcriptome data of Group AOSC compared to Group CD-AOSC, highlighting macrophage and Th1 cell activation. (B)-(C) GSEA enrichment plots of GSE5960 and GSE1925. (D) The heatmap displayed significant positive correlations between IL-1β, TNF, IL-18, IFN-γ, NFκB1, IL18RAP, and NOS1 with macrophage and Type 1 T helper cell activation (^*^P<0.05, ^**^P<0.01, ^***^P<0.001). Th1 cell: type I helper cell; AOSC: acute obstructive suppurative cholangitis; CD-AOSC: catecholamine-dependent acute obstructive suppurative cholangitis; IFNG: interferon-γ; TNF: tumor necrosis factor; IL-1B: interleukin-1β; IL18RAP: interleukin 18 receptor accessory protein; NFκB1: nuclear factor kappa B subunit 1; NOS1: nitric oxide synthase 1.

For identifying the immune cells that significantly participated in the CD-AOSC, the GSEA were performed. “GSE1925_3H_VS_24H_IFNG_STIM_ MACROPHAGE_UP” and “GSE5960_TH1_VS_ ANERGIC_TH1_UP” were the common pathways that enriched in Group AOSC than CD-AOSC (Figure 4A-C) and in Group CD-AOSC than CC (Supplementary Figure S8A–C). These two pathways involved macrophage and Th1 cells, which were important to “inflammatory bowel disease pathway” mentioned in KEGG enrichment analysis (Supplementary Figure S7B).

We further used the GSV package to calculate the enrichment scores of various immune cells in our transcriptome dataset to consolidate the findings in GSEA analysis. The GSVA score of Th1 and macrophages were significantly higher in Group AOSC compared to Group CD-AOSC (Supplementary Figure S8E), though there was no statistical difference between Groups CD-AOSC and CC (Supplementary Figure S8F). There were also a significant positive Pearson correlation of immune cell GSVA score between type I helper cells (Th1) and macrophages among the three groups (Supplementary Figure S8D). IL1B, TNF, IL18, IFN-γ, NF-κB1, IL18RAP, and nitric oxide synthase 1 (NOS1) genes were significantly positively correlated with Th1 and macrophage stimulation (Figure 4D).

### The expressing pattern in the upstream of IL-1β and IL-18 were different from those downstream of IL-1β and IL-18 indicated some unknown pro-inflammatory cytokines caused the CD-AOSC

PCR analysis was performed for molecules downstream of the LPS/ Toll-like receptor 4 (TLR4) signaling pathway and upstream of proinflammatory cytokines such as IL-1β and IL-18 (Supplementary Figure S9A) in preoperative patient blood.

These molecules included IRAK1, IRAK4, JUN, CHUK, IKBKB, TRAF3, TBK1, and IRF3. Expression levels were significantly higher in Group AOSC and Group CD-AOSC compared with Group CC. No significant difference was observed between Group AOSC and Group CD-AOSC (Supplementary Figure S9B-I).

This expression pattern differed from the serum levels of IFN-γ, IL-1β, and IL-18 (Supplementary Figure S10, Table 2). The latter matched the expression patterns of adipose tissue endotoxin levels and inflammatory cell infiltration in adipose tissue (Figure 2B, G, I).

### Reduced inflammation caused by IL-1β inhibitor slows the endotoxin elimination in rat’s adipose tissue

**Figure 5:**
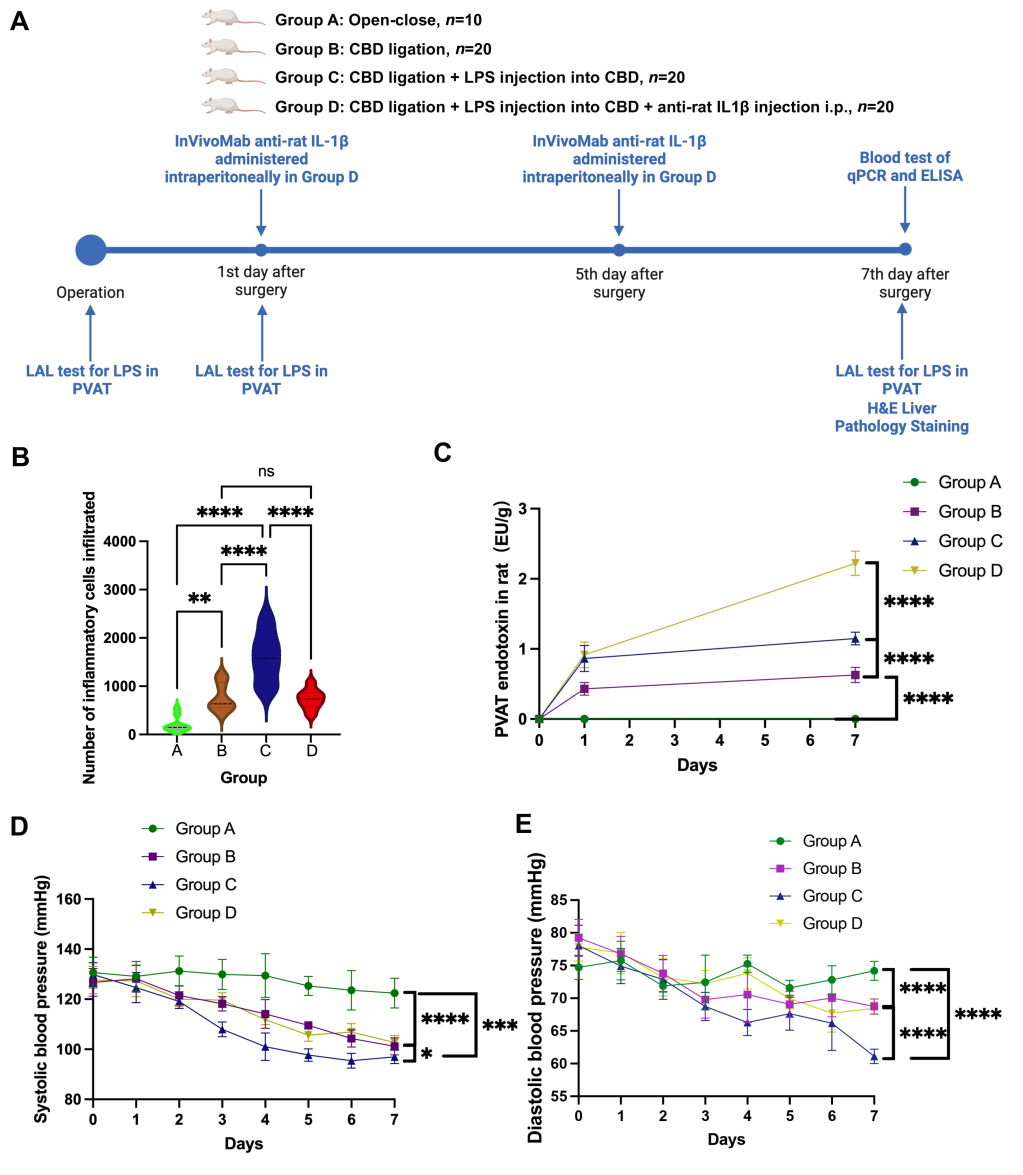
IL-1β inhibitor alleviates inflammation in a rat cholangitis model by reducing endotoxin clearance from PVAT. (A) Experimental design flowchart for the rat model. (B) H&E staining (Figure S9-12) showed significantly higher inflammatory cell infiltration in the portal area of Group C compared to Groups A, B, and D, with no significant difference between Groups B and D. (C) Endotoxin levels in PVAT, measured on days 0, 1, and 7 *via* LAL assay, showed a continuous increase in Groups B, C, and D. On day 7, endotoxin levels were highest in Group D, significantly higher than in Groups A, B, and C, while Group C had significantly higher levels than Groups B and A. (D-E) Systolic (SBP) and diastolic blood pressure (DBP) remained normal in Group A but gradually declined post-surgery in other groups. By day 7, SBP in Group A was significantly higher than in Groups B, C, and D, with no significant difference between Groups B and D. DBP followed a similar trend. (^*^P<0.05, ^**^P<0.01, ^***^P<0.001, ^****^P<0.0001). CBD: common bile duct; LPS: lipopolysaccharides; IL-1β: interleukin-1β; qPCR: quantitative polymerase chain reaction; ELISA: enzyme-linked immunosorbent assay; LAL: limulus amoebocyte lysate; PVAT: perivascular adipose tissue.

To verify whether reduced inflammation could lead to endotoxin retention and IL18 pathway activation, we conducted the experiment in a rat model. According to the data in Table 2, we used IL-1β inhibitor to reduce the inflammation in rat cholangitis model (Figure 5A). The detail of the surgery and experiment were described in the materials and methods.

All 10 rats in the Group A survived. In the Group B, forteen survived after 7 days after surgery, with three died on the second day, and three died on the third day. In the group C, twelve survived after 7 days, with three died on the first day, and five died on the fourth day after surgery. In the Group D, forteen survived after 7 days, with 4 died on the second day and 2 died on the third day.

Seven days after surgery, H&E-stained liver tissue sections were obtained from three euthanized rats per group for histological analysis (Figures S11-S14).

The number of inflammatory cells in the portal area was significantly higher in Group C (1603.0 ± 519.0) than in Group A (204.4 ± 166.3), Group B (765.4 ± 289.4), and Group D (732.7 ± 206.8). No significant difference was observed between Group B and Group D (Figure 5B).

The endotoxin levels in rats’ PVAT in the different groups tested with the LAL assay showed a continuous increase in Group B, C, and D. On day 7, the endotoxin level was significantly higher in Group D (2.2 ± 0.2 EU/g) compared with the other three groups (*P* < 0.0001). The level in Group C (1.2 ± 0.1 EU/g) was significantly higher than that in Group B (0.7 ± 0.1 EU/g) and Group A (*P* < 0.0001) (Figure 5C).

The systolic and diastolic blood pressure of the rats in Group A remained in the normal range and gradually decreased over time in the other groups after the surgery. On the 7th day after surgery, systolic blood pressure in Group A (122.4 ± 6.0 mmHg) was significantly higher than in Group B (101.2 ± 3.3 mmHg), Group C (97.0 ± 2.6 mmHg), and Group D (102.7 ± 2.7 mmHg). Group B and Group D showed significantly higher systolic blood pressure values than Group C (Figure 5D). The diastolic blood pressure on the 7th day after surgery showed a similar pattern to the systolic blood pressure (Figure 5E).

**Figure 6:**
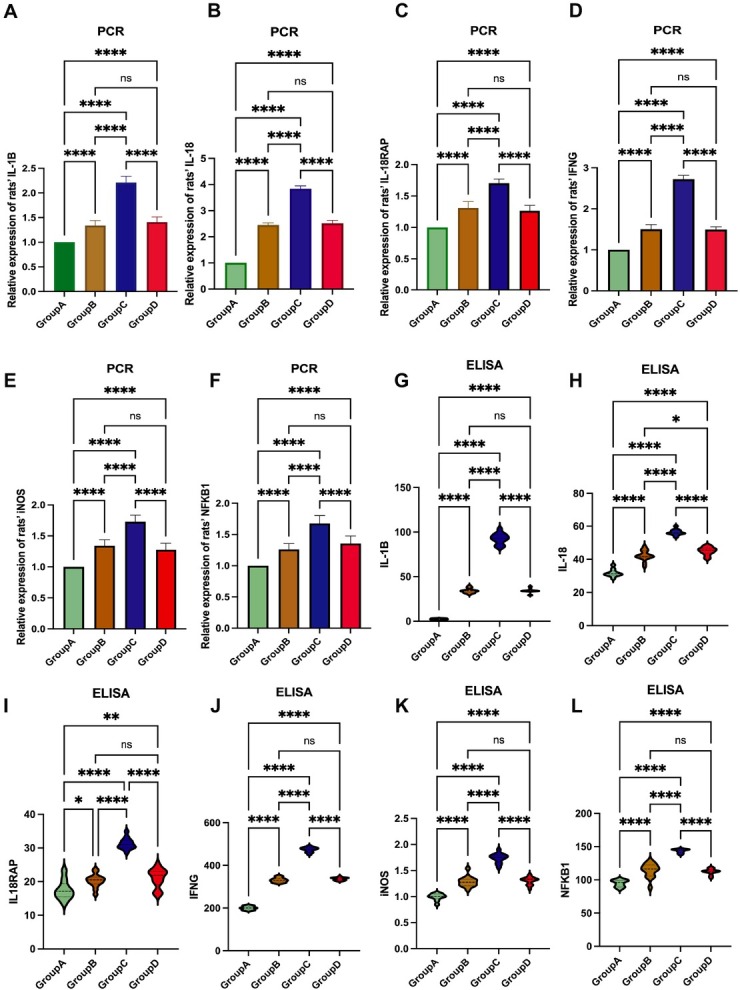
IL18 pathway expression levels align with previously observed inflammation severity and blood pressure changes in rats. (A-F) PCR analysis of IL-1β, IL-18, IL-18RAP, IFNG, iNOS, and NFκB1 expression in rat blood showed significantly higher expression in Group C compared to Groups A, B, and D, with no significant difference between Groups B and D. (G-L) ELISA analysis for IL-1β, IL-18, IL-18RAP, IFN-γ, iNOS, and NF-κB1 protein levels in rat blood revealed significantly higher levels in Group C compared to Groups A, B, and D, with no significant difference between Groups B and D. (^*^P <0.05, ^**^P <0.01, ^****^P < 0.0001). PCR: polymerase chain reaction; ELISA: enzyme-linked immunosorbent assay; IL-1B: interleukin-1B; IL-18RAP: interleukin 18 receptor accessory protein; IFNG: interferon-γ; iNOS: inducible nitric oxide synthase; NFκB1: nuclear factor kappa-B 1.

On day 7 post-euthanasia, qPCR analysis of rat blood revealed significant increase in the expression level of IL-1β, IL-18, IL-18RAP, IFN-γ, iNOS, and NF-κB1 expression levels in Group C compared to Group A, B, and D (*P* < 0.0001). In contrast, there were no significant differences in these expression levels between Groups B and D (Figure 6A-F).

Similarly, ELISA results indicated that the concentrations of IL- 1β, IL- 18, IL- 18RAP, IFN-γ, iNOS, and NF-κB1 were significantly elevated in Group C relative to Group A, B, and D (*P* < 0.0001), with no significant differences observed between Group B and D ( Figure 6G-L).

### The duration of continuous rise in serum levels of molecules in the IL-1β/IL-18 pathway is coincided with the period of hypotension in CD-AOSC patients

**Figure 7:**
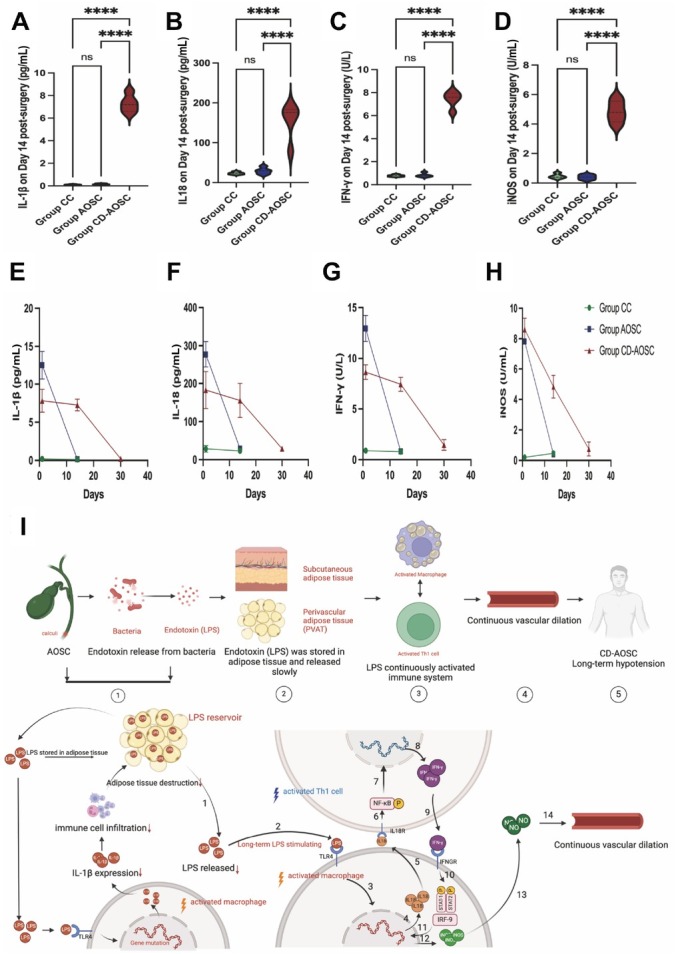
IL-18/ IFN-γ/NF-κB1/iNOS contribute to prolonged hypotension in CD-AOSC. (A)-(D) Blood levels of IL-1β, IL-18, IFN-γ, and iNOS on day 14 post-surgery. These molecules were significantly elevated in Group CD-AOSC compared to Groups CC and AOSC, while no significant differences were observed between CC and AOSC, both of which remained within the normal range. (E)- (H) Thirty-day trends of IL-1β, IL-18, IFN-γ, and iNOS level change in all groups, showing a gradual decrease in Group CD-AOSC, which normalized as blood pressure stabilized. (Group CC and AOSC only tested on the day before surgery and the 14th day while Group CD-AOSC tested on all the 3 time points, 0, 14th, and 30th day.) (I) Proposed mechanism of CD-AOSC: Lower IL-1β levels reduce adipose tissue inflammation, allowing endotoxin accumulation. The slow release of endotoxin activates IL-18/IL-18RAP/ IFN-γ/NF-κB1, leading to sustained hypotension. (^****^P < 0.0001). Th1 cell: type I helper cell; CC: chronic cholecystitis; AOSC: acute obstructive suppurative cholangitis; CD-AOSC: catecholamine-dependent acute obstructive suppurative cholangitis; IL-1B: interleukin-1B; IL-18RAP: interleukin 18 receptor accessory protein; IFN-γ: interferon-γ; iNOS: inducible nitric oxide synthase; NFκB: nuclear factor kappa-B; LPS: lipopolysaccharides.

Blood samples (*n* = 5 in each group) were collected from the patients from Group CC, AOSC, and CD-AOSC 14 and 30 days post-operation, as outlined in the Methods section. On the 14th day after surgery, the serum levels of IL-1β, IL-18, IFN-γ, and iNOS were significantly elevated in Group CD-AOSC compared to Group CC and AOSC. No significant differences were observed between Group CC and AOSC, with their levels remaining within the normal range (Figure 7A-D).

The temporal trend from day 1 to day 30 indicated that the serum levels of IL-1β, IL-18, IFN-γ, and iNOS remained elevated for most of the period when the CD-AOSC patients were in a hypotensive state. These levels returned to normal upon normalization of blood pressure and cessation of the catecholamine pump (Figure 7E-H).

## Discussion

### The common etiology of long-term hypotension in literature review

Our research into CD-AOSC highlights it as a rare condition, characterized by extended hypotension, often persisting for about 30 days.

This prolonged hypotensive state could arise from multiple factors. Literature review, including Chuang *et al*. on anaphylaxis-induced refractory hypotension and Li *et al*. on aortocaval compression syndrome, offers insights into varied hypotension scenarios.^[3,4]^ Memon *et al*. discussed orthostatic hypotension (OH) as another variant.^[5]^ Hojo *et al*. find that the influence of general anesthesia in patients with a history of hypertension or those on angiotensin receptor blockers (ARBs) can cause refractory hypotension during and after the surgery.^[6]^ However, these cases do not align with the etiology of CD-AOSC in our study, where patients showed almost normal cognitive function, cortisol levels and near-normal inflammatory markers post-biliary drainage surgery, eliminating these potential causes for prolonged hypotension.

### Our findings in CD-AOSC

Endotoxin initiates sepsis-related inflammation *via* the TLR4 which requires lipopolysaccharide binding protein (LBP) for activation.^[7]^ TLR4- myeloid differentiation primary response protein 88 (MyD88) and TLR4-Toll/IL-1 receptor domain-containing adaptor-inducing interferon-β (TRIF) pathways are involved in these mechanisms, highlighting the complexity of the role of endotoxin in sepsis pathogenesis and the challenges in recognizing it for precise therapeutic interventions.

In our study, the adipose tissues of the CD-AOSC group exhibited significantly higher endotoxin levels compared to Group CC and AOSC, which slowly decrease in the subcutaneous liquefication adipose tissue after emergent surgery. This suggests that adipose tissue acts as a reservoir that impedes recovery and contributing to persistent hypotension. PVAT endotoxin was predictive of blood pressure recovery in sepsis in our multivariable regression, LASSO regression, and random forest models. It was significantly positively correlated with blood pressure recovery time, underscoring the important role of PVAT endotoxin in CD-AOSC.

The reduced inflammatory levels observed in the adipose tissue of CD-AOSC patients, both at the tissue and transcriptome levels, align with the pattern of inflammatory markers such as HBP, IFN-γ, IL-1β, IL-6, and IL-18. However, the expression pattern of molecules downstream of the LPS/TLR4 signaling pathway and upstream of pro-inflammatory cytokines, including IRAK1/4, JUN, CHUK, TRAF3, TBK1, IRF3, and IKBKB, displayed a different trend in the three groups. This discrepancy suggests the presence of a pro-inflammatory cytokine whose expression or function is diminished due to gene mutations, epigenetic modifications, or abnormalities in RNA interference. Based on changes in clinical indicators, we evaluated the effect of an IL-1β inhibitor and found that it effectively promoted endotoxin accumulation in adipose tissue and attenuated inflammatory marker levels in a rat model of cholangitis.

In patients with CD-AOSC, gradual adipose tissue degradation results in the continuous release of endotoxin, contributing to persistent hypotension. This endotoxin drives macrophage and Th1 activation, with increased IL-18/IL-18R signaling. IL-18, a pro-inflammatory cytokine, correlates with the severity of sepsis through IL-18R interaction,^[8,9]^ which activates Th1 cells and induces IFN-γ production *via* NF-κB.^[10,11]^ IL18RAP enhances IL-18/IL-18R binding, further boosting IFN-γ production, seen in sepsis.^[12,13]^ IFN-γ and TNF-α stimulate iNOS in macrophages, producing nitric oxide (NO), which triggers vasodilation and hypotension by activating soluble guanylyl cyclase (sGC) in arterial endothelial cells.^[14, 15, 16, 17, 18]^ The mechanism was summarized in the Figure 7I.

### The potential management of cytokine storm

Cytokine storm is a severe immune response where the body’s pro-inflammatory mechanisms far outweigh its anti-inflammatory controls. This phenomenon often leads to systemic inflammation and multi-organ failure if left untreated. Cytokine storms can result from various triggers, such as infections, autoimmune diseases, or therapeutic interventions like chimeric antigen receptor T-cell (CAR-T) therapy. The failure to regulate cytokine production leads to a hyperactive immune response characterized by excessive levels of pro-inflammatory cytokines, such as IFN-γ, IL-1, IL-6, and TNF. Inappropriate activation of immune cells, particularly Th1 cells, macrophages, and cytotoxic T lymphocytes (CTLs), drives this excessive response, causing tissue damage and organ dysfunction.^[19, 20, 21, 22]^

Research has demonstrated that cytokine storms are associated with various diseases, including sepsis and hemophagocytic lymphohistiocytosis (HLH). Targeted cytokine therapies have shown potential benefits. Emapalumab, an antibody against IFN-γ, has been approved for treating cytokine storms in primary HLH, while the IL-1 receptor antagonist anakinra has proven effective in managing certain cytokine storms.^[23, 24, 25]^ De Benedetti *et al*. reported that canakinumab, an antibody specific to IL-1β, binds exclusively to human IL-1β and not to mouse IL-1β, limiting its use in experimental studies on cytokine storms in inflammatory bowel disease (IBD).^[26]^ Additionally, TNF, a key pro-inflammatory cytokine, has been implicated in cytokine storm progression, but anti-TNF therapies have shown limited effectiveness in septic patients due to potential risks.^[25]^

Emerging studies also highlight the role of IL-18 in cytokine storm diseases, particularly in conditions like macrophage activation syndrome (MAS). IL-18, activated through inflammasomes, plays a central role in stimulating Th1 responses by promoting IFN-γ production. Elevated serum levels of IL-18 have been identified as biomarkers of disease severity in patients with MAS, and therapies targeting IL- 18, such as Tadekinig alfa, are under investigation.^[27, 28, 29, 30]^

One of the key challenges in managing cytokine storms lies in balancing immune suppression with pathogen control. In cases like sepsis, where endotoxin from Gram-negative bacteria triggers systemic inflammation, rapid detection and targeted therapy are critical to preventing organ failure.^[31, 32, 33]^ Despite the progress in understanding the mechanisms behind cytokine storms, several challenges remain in identifying optimal therapeutic interventions. While targeted cytokine blockade has shown promise, more research is needed to understand the full spectrum of immune dysregulation in these storms and to develop therapies that mitigate the risks of both excessive inflammation and immune suppression.

In sepsis-associated cytokine storms, the specific immune cells and cytokines responsible for pathological inflammation remain poorly understood. Although antibiotics are the primary treatment, monoclonal antibodies and cytokine removal strategies have shown limited effectiveness in clinical trials.^[34]^ Factors such as treatment timing and host or pathogen characteristics may influence these outcomes. Interestingly, a reanalysis of interleukin-1β blockade in sepsis demonstrated benefits for patients with elevated ferritin levels.^[35]^ Oh *et al*. reported that mAb 7F IgG, a novel IL-1β-specific antibody, effectively blocks the IL-1β pathway and exhibits anti-colitis activity in IBD mouse models.^[36]^ Additionally, the IL-1R antagonist anakinra has been evaluated in clinical trials for IBD to assess its impact on macroscopic and histological changes, inflammatory cell infiltration, and oxidative stress in colitis.^[37]^

### The potential role of adipose tissue could be exploited as target for cytokine storm in sepsis treatment

Bacterial infections in organs are often accompanied by adipose tissue edema. Our findings suggest that endotoxins can transiently accumulate in adipose tissue, attracting inflammatory cells and modulating the local immune response. When pro- and anti-inflammatory pathways remain balanced, symptoms subside as endotoxins are cleared, facilitating recovery. However, in CD-AOSC patients, endotoxin clearance is impaired, inflammatory cell infiltration is reduced, and recovery is prolonged, contributing to sustained hypotension.

These observations highlight the potential role of adipose tissue as a reservoir for endotoxins. A deeper understanding of this mechanism may inform the development of precision therapies targeting key cytokines—such as IL-1β and IL-18—or downstream effectors to mitigate cytokine storms in severe AOSC patients with sepsis and reduce mortality.

Numerous studies on the obesity paradox suggest that obesity is inversely related to mortality in patients with septic shock, indicating that obesity may reduce mortality in such cases.^[38, 39, 40, 41, 42, 43]^ However, the underlying mechanisms remain poorly understood. This study suggests that adipose tissue may temporarily sequester endotoxins during septic shock, providing a potential explanation for this paradox.

Our findings suggest that in a subset of severe sepsis patients driven by endotoxemia, early targeting of IL-1β may represent a rational therapeutic strategy to slow endotoxin release and attenuate sepsis progression, potentially reducing the severity of the cytokine storm and associated mortality, albeit at the cost of a prolonged recovery period. We refer to this strategy of trading time for a reduction in inflammation severity as “Hours Exchanged Against (severity) Loss (HEAL).” Based on this HEAL hypothesis, obese patients without diabetes may derive a survival benefit from early IL-1β-targeted intervention during the initial stages of severe sepsis of cholangitis.

This study is limited by the relatively small number of cases, and future multicenter prospective studies with larger and more balanced cohorts are needed to validate our findings. Further investigation is also warranted to explore potential therapeutic targets within downstream pathways of IL-18. Although the mechanisms underlying delayed endotoxin clearance remain incompletely understood, our findings offer novel insights into the selection and timing of therapeutic interventions for patients with severe cholangitis.

## Conclusion

Persistent hypotension in CD-AOSC appears to result from sustained activation of the IL-18/IL-18RAP pathway, driven by prolonged endotoxin release from adipose tissue. These findings highlight the potential of “HEAL” strategy, in which early modulation of endotoxin release from adipose tissue by IL-1β inhibition during the initial phase of sepsis in cholangitis may improve patient survival.

## Supplementary Information

Supplementary materials are only available at the official site of the journal (www.intern-med.com).

## Supplementary Material

Supplementary Material Details
