# Supplementary material for: Endotoxin retention in adipose tissue leading to persistent hypotension in catecholamine-dependent acute obstructive suppurative cholangitis
Source: J Transl Int Med. 2025 Dec 22;13(6):537–58. doi: 10.1515/jtim-2025-0053 (PMC12721365; doi:10.1515/jtim-2025-0053)
Supplement: Supplementary file 1 — Supplementary Material Details [file jtim-2025-0053_sm.pdf]

Endotoxin Retention in Adipose Tissue Leading to Persistent Hypotension in Catecholamine-Dependent Acute Obstructive Suppurative Cholangitis

Running title: Long-Term Hypotension Mechanism in CD-AOSC

Kun Li[1,3,†], Sibo Zhu[4, †], Chun Ye[1], Yue Ding[1], Jiangxi Liu[1], Yufeng Wang[1], Zhiyong Wang[5], Wenfeng Li[5], Dong-Hua Yang[6], Xiuyan Wang[7], Rui Lin[1,5,#], Baomin Shi[1,2,#]

1. General Surgery Department, Tongji Hospital, School of Medicine, Tongji University, Shanghai, 200065, China
2. Department of General Surgery, Xinhua Hospital Affiliated to Shanghai Jiao Tong University School of Medicine, Shanghai, 200092, China
3. Department of General Surgery, Tongren Hospital, Shanghai Jiao Tong University School of Medicine, Shanghai, 200336, China
4. MOE Key Laboratory of Contemporary Anthropology, School of Life Sciences, Fudan University, Shanghai, 200438, China
5. Surgery Department, Chongyi County People's Hospital, Jiangxi Province, 341300, China
6. Administrative Dean, New York College of Traditional Chinese Medicine, 200 Old Country Rd, Suite 500 Mineola, NY, 11501, USA
7. Ultrasound Department, Tongji Hospital, School of Medicine, Tongji University, Shanghai, 200065, China

Table of contents

Supplementary Figures 3

Supplementary Table 21

Supplementary Figures

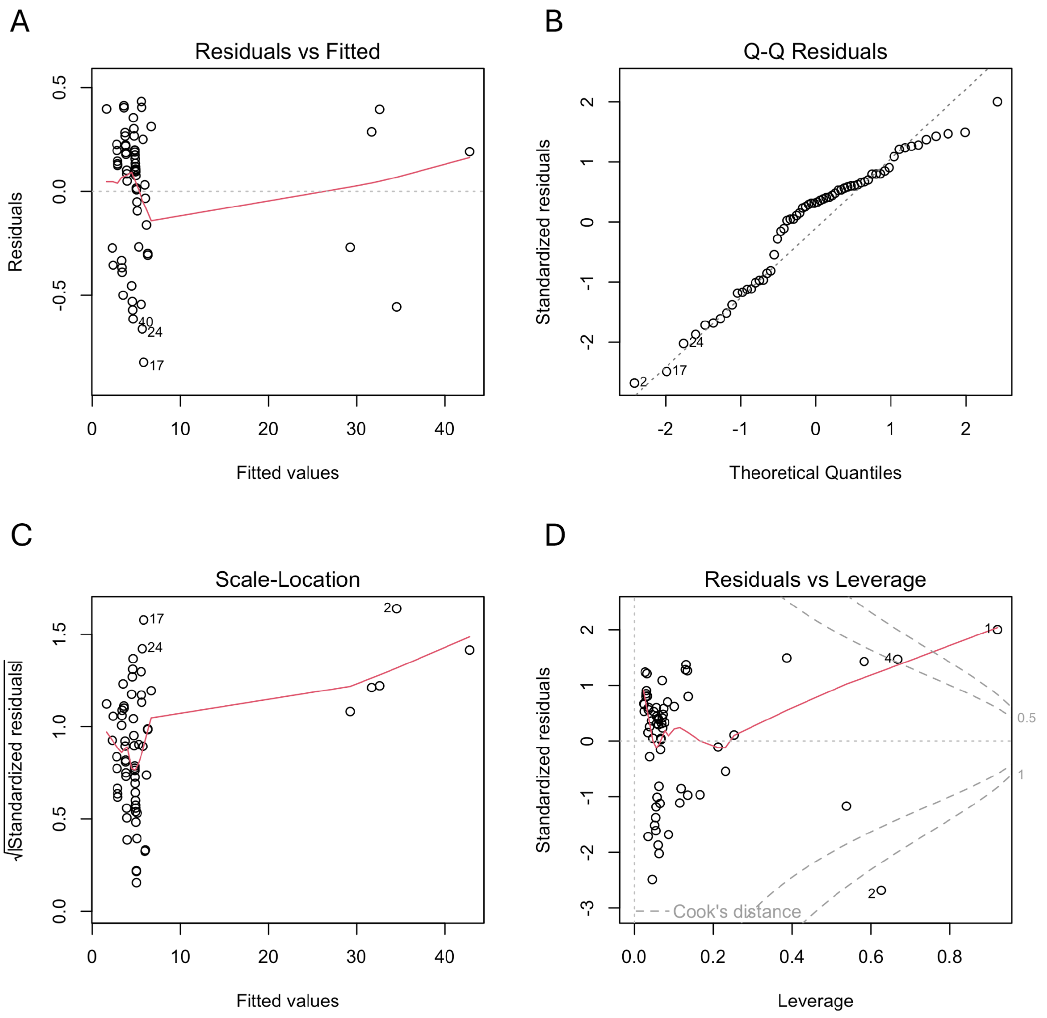

Fig.S1 Diagnostic plots of the final multivariable linear regression model.

(A) Residuals vs Fitted: assesses linearity and homoscedasticity.

(B) Normal Q-Q plot: evaluates the normality of residuals.

(C) Scale-Location: checks for equal variance across fitted values.

(D) Residuals vs Leverage: detects influential data points.

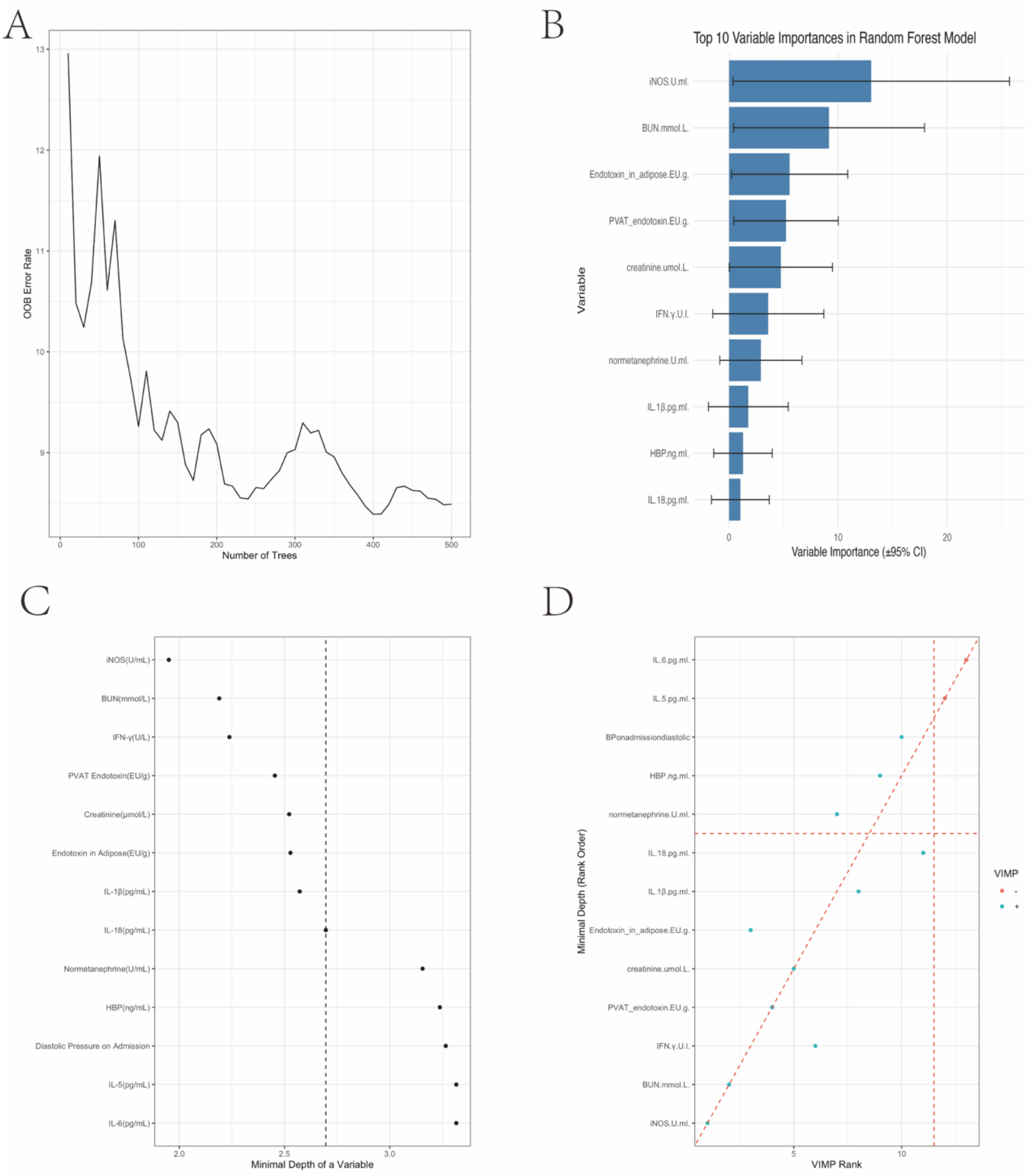

Fig. S2 Random forest model analysis.

(A) Out-of-bag (OOB) error was used to estimate generalization error across increasing numbers of trees.

(B) Variable importance (VIMP) was calculated to rank features contributing most to model accuracy. The mean and standard error (SE) of each variable's VIMP across replications were used to derive 95% confidence intervals. The top

10 variables were visualized using bar plots with error bars representing ±1.96×SE.

(C) Minimal depth was assessed to determine how early variables were selected in tree splits.

(D) A combined analysis of VIMP and minimal depth was used to identify key predictive features.

CBDE: Common bile duct exploration; LCBDE: Laparoscopic common bile duct exploration; HBP: Heparin binding protein; BUN: Blood urea nitrogen; DBil: Direct bilirubin; TBil: Total bilirubin; CRP: C-reactive protein; ALT: Alanine aminotransferase; AST: Aspartate aminotransferase; BPonadmissiondiastolic: Diastolic blood pressure on admission; BPonadmissionsystolic: Systolic blood pressure on admission.

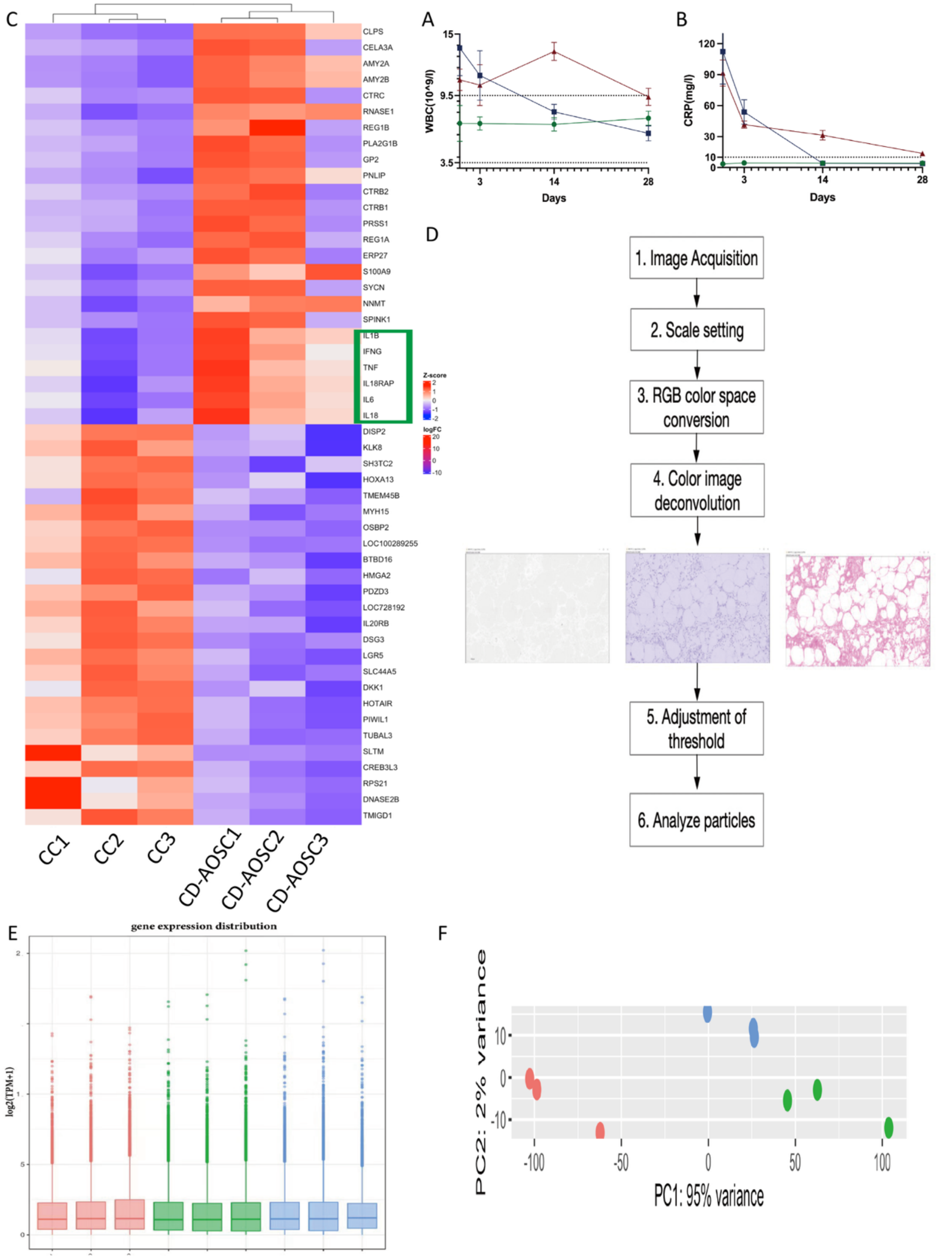

Fig.S3 Transcriptome analysis of PVAT and blood samples, and quantification method of inflammatory cells in H&E-stained images.

(A) – (B) The temporal trends of blood WBC and CRP levels were analyzed at admission and on postoperative days 3, 14, and 28. In the figure, the horizontal

dotted line marks the normal reference range for WBC and CRP.

(C) The top 25 upregulated and downregulated differentially expressed genes (DEGs) in PVAT between Group CD-AOSC and CC, with the most relevant genes highlighted in the green box.

(D) Workflow for cell counting analysis in H&E-stained slices using the Colour_Deconvolution2 extension in ImageJ.

(E) Gene expression distribution from transcriptome analysis of blood samples from different patient groups.

(F) Principal Component Analysis (PCA) of blood transcriptome data from different patient groups indicated different clusters for 3 different patient groups.

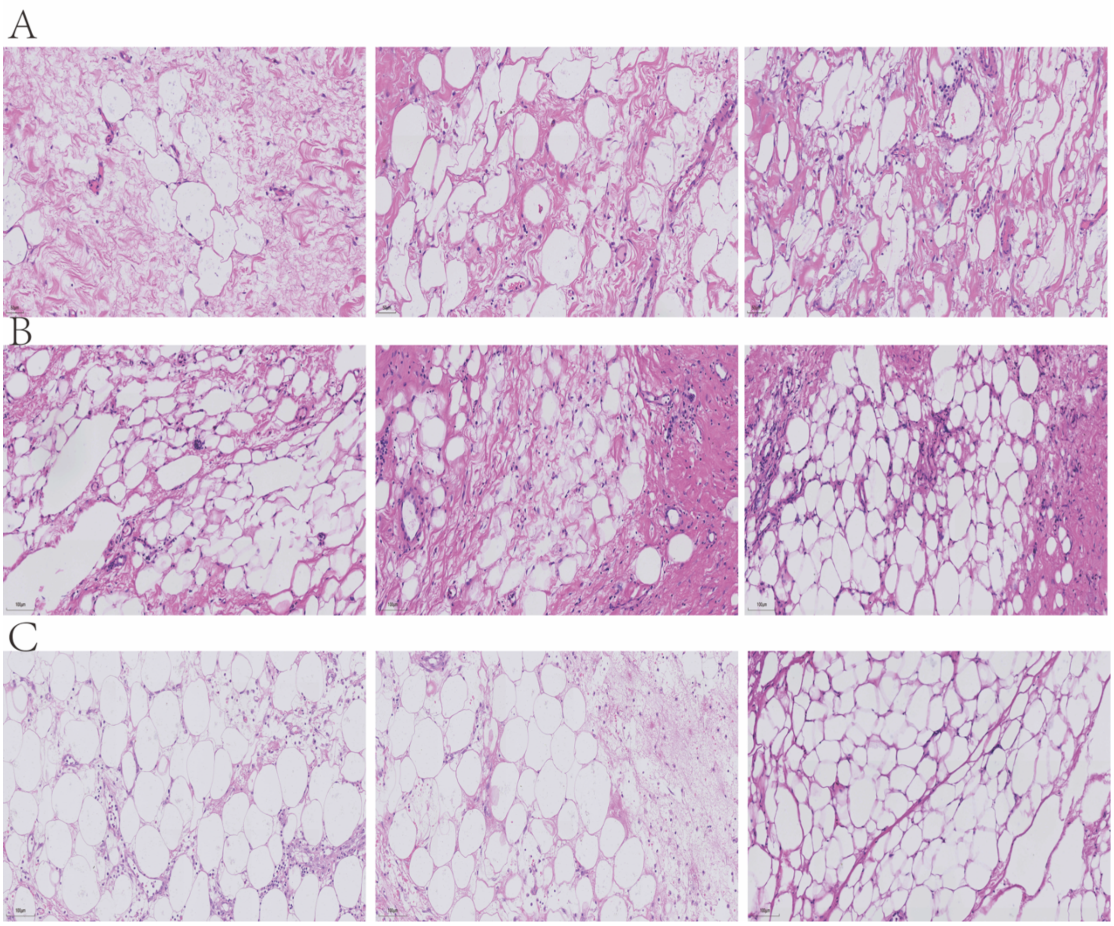

Fig.S4 H&E staining of PVAT pathological slice images. (A)-(C) show three different slices from three different patients' samples in Group CC.

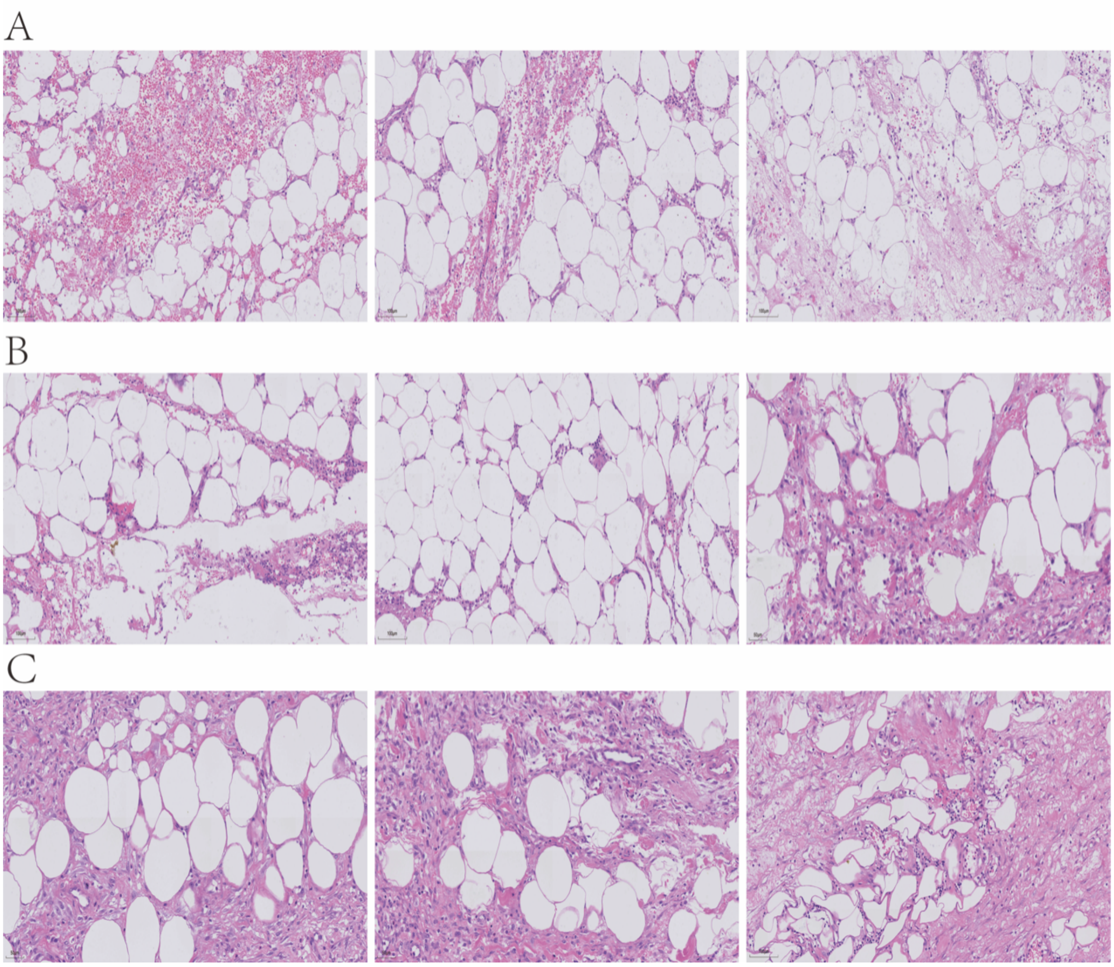

Fig.S5 H&E staining of PVAT pathological slice images. (A)-(C) show three different slices from three different patients' samples in Group AOSC.

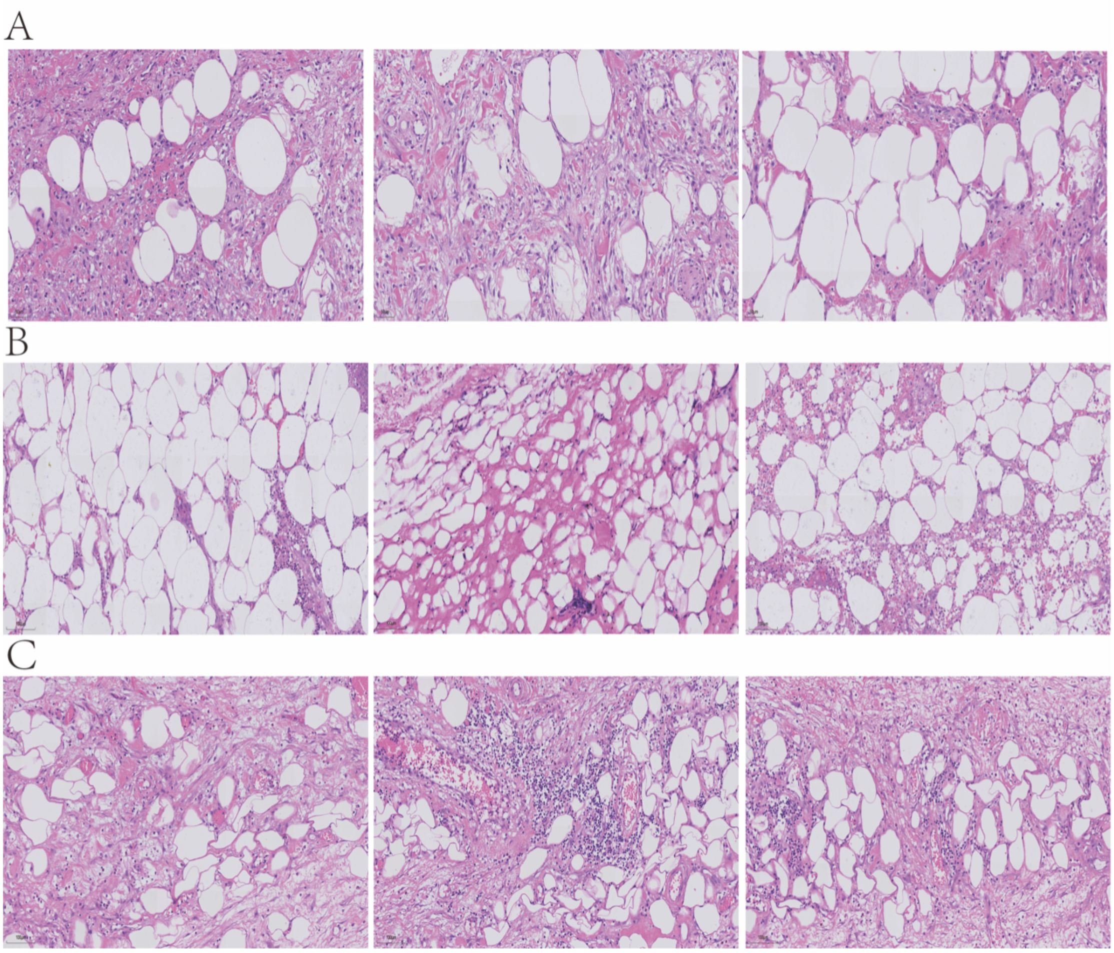

Fig.S6 H&E staining of PVAT slice images. (A)-(C) show three different slices from three different patients' samples in Group CD-AOSC.

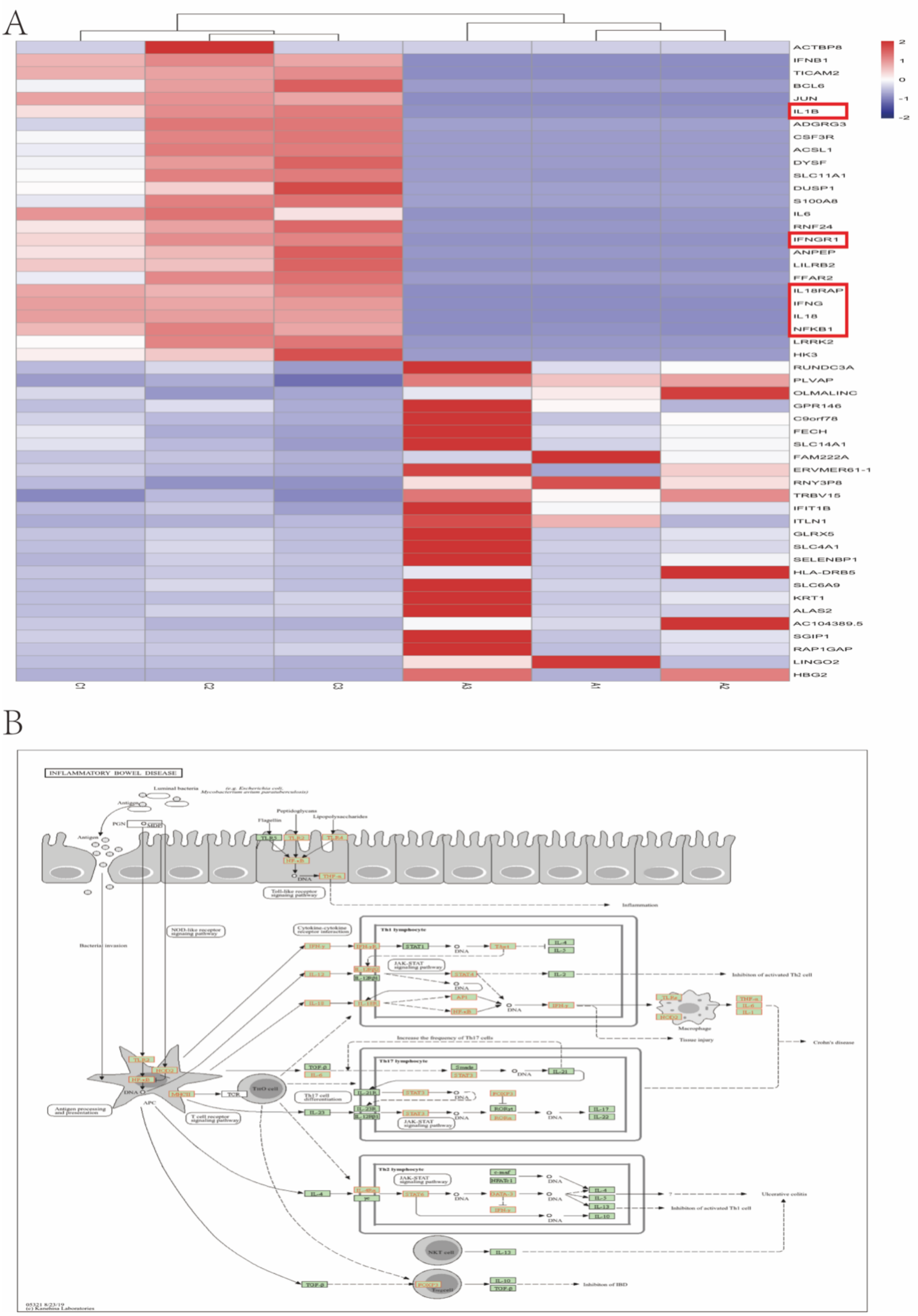

Fig.S7 The enriched KEGG pathway of 'inflammatory bowel disease' contained the most molecules found in differentially expressed genes among the three groups. (A) Analysis of differentially expressed genes (DEGs) of patients' blood revealed that IL1B, IFNGR1, IL18RAP, IFNG, IL18, and NFKB1 were significantly

upregulated in Group CD-AOSC compared to Group CC (highlighted in red boxes).
(B) KEGG pathway analysis identified the "inflammatory bowel disease" pathway as commonly enriched between Group CD-AOSC and CC, Group CD-AOSC and AOSC. The IL18/IL18R -> NF-κB -> IFN-γ -> TNF-α/IL-6 pathway may play a significant role in Group CD-AOSC.

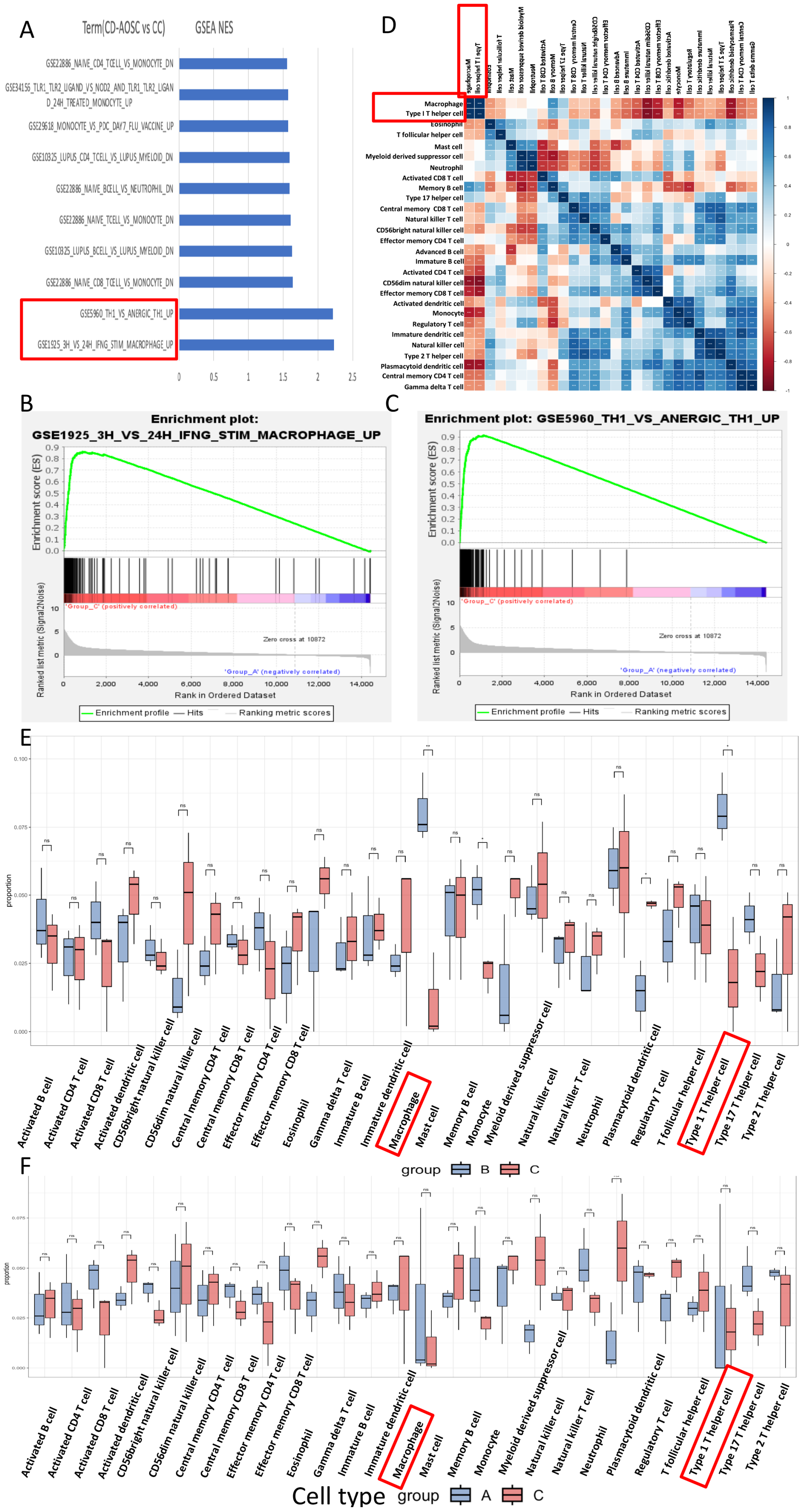
A
Term(CD-AOSC vs CC)
GSEA NES
GSE22886_NAIVE_CD4_TCELL_VS_MONOCYTE_DN
GSE34156_TLR1_TLR2_LIGAND_VS_NOD2_AND_TLR1_TLR2_LIGAN D_24H_TREATED_MONOCYTE_UP
GSE29618_MONOCYTE_VS_PDC_DAY7_FLU_VACCINE_UP
GSE10325_LUPUS_CD4_TCELL_VS_LUPUS_MYELOID_DN
GSE22886_NAIVE_BCELL_VS_NEUTROPHIL_DN
GSE22886_NAIVE_TCELL_VS_MONOCYTE_DN
GSE10325_LUPUS_BCELL_VS_LUPUS_MYELOID_DN
GSE22886_NAIVE_CD8_TCELL_VS_MONOCYTE_DN
GSE5960_TH1_VS_ANERGIC_TH1_UP
GSE1925_3H_VS_24H_IFNG_STIM_MACROPHAGE_UP
D
Macrophage
Type I T helper cell
Eosinophil
T follicular helper cell
Mast cell
Myeloid derived suppressor cell
Neutrophil
Activated CD8 T cell
Memory B cell
Type 17 helper cell
Central memory CD8 T cell
Natural killer T cell
CD56bright natural killer cell
Effector memory CD4 T cell
Advanced B cell
Immature B cell
Activated CD4 T cell
CD56dim natural killer cell
Effector memory CD8 T cell
Activated dendritic cell
Monocyte
Regulatory T cell
Immature dendritic cell
Natural killer cell
Type 2 T helper cell
Plasmacytoid dendritic cell
Central memory CD4 T cell
Gamma delta T cell
B
Enrichment plot:
GSE1925_3H_VS_24H_IFNG_STIM_MACROPHAGE_UP
Enrichment score (ES)
'Group_C' (positively correlated)
Zero cross at 10872
'Group_A' (negatively correlated)
Ranked list metric (Signal2Noise)
Rank in Ordered Dataset
Enrichment profile — Hits — Ranking metric scores
C
Enrichment plot: GSE5960_TH1_VS_ANERGIC_TH1_UP
Enrichment score (ES)
'Group_C' (positively correlated)
Zero cross at 10872
'Group_A' (negatively correlated)
Ranked list metric (Signal2Noise)
Rank in Ordered Dataset
Enrichment profile — Hits — Ranking metric scores
E
proportion
Activated B cell
Activated CD4 T cell
Activated CD8 T cell
Activated dendritic cell
CD56bright natural killer cell
CD56dim natural killer cell
Central memory CD4 T cell
Central memory CD8 T cell
Effector memory CD4 T cell
Effector memory CD8 T cell
Eosinophil
Gamma delta T cell
Immature B cell
Immature dendritic cell
Macrophage
Mast cell
Memory B cell
Monocyte
Myeloid derived suppressor cell
Natural killer cell
Natural killer T cell
Neutrophil
Plasmacytoid dendritic cell
Regulatory T cell
T follicular helper cell
Type 1 T helper cell
Type 17 T helper cell
Type 2 T helper cell
group B C
F
proportion
Cell type
group A C

Fig.S8 GSEA analysis and GSVA immune score of transcriptome data from patients' blood samples in different groups

(A) The top 10 GSEA enriched gene sets upregulated in Group CD-AOSC compared to Group CC.

(B-C) Enrichment plot of 'GSE1925' and 'GSE5960' between Group CD-AOSC and CC.

(D) The heatmap of Pearson correlation, using the immune cells GSVA scores, revealed type I helper cell (Th1) were significantly positive correlating with macrophage cells (correlation coefficient was 0.998, *P* = 1.24*10-37).

(E) GSVA enrichment scores for various immune cells from transcriptome data showed the score of macrophage and Type 1 helper T cell were significantly higher in Group AOSC compared to Group CD-AOSC.

(F) GSVA enrichment scores for various immune cells from transcriptome data showed no significant differences in the score of different immune cell types in Group CD-AOSC compared to Group CC.

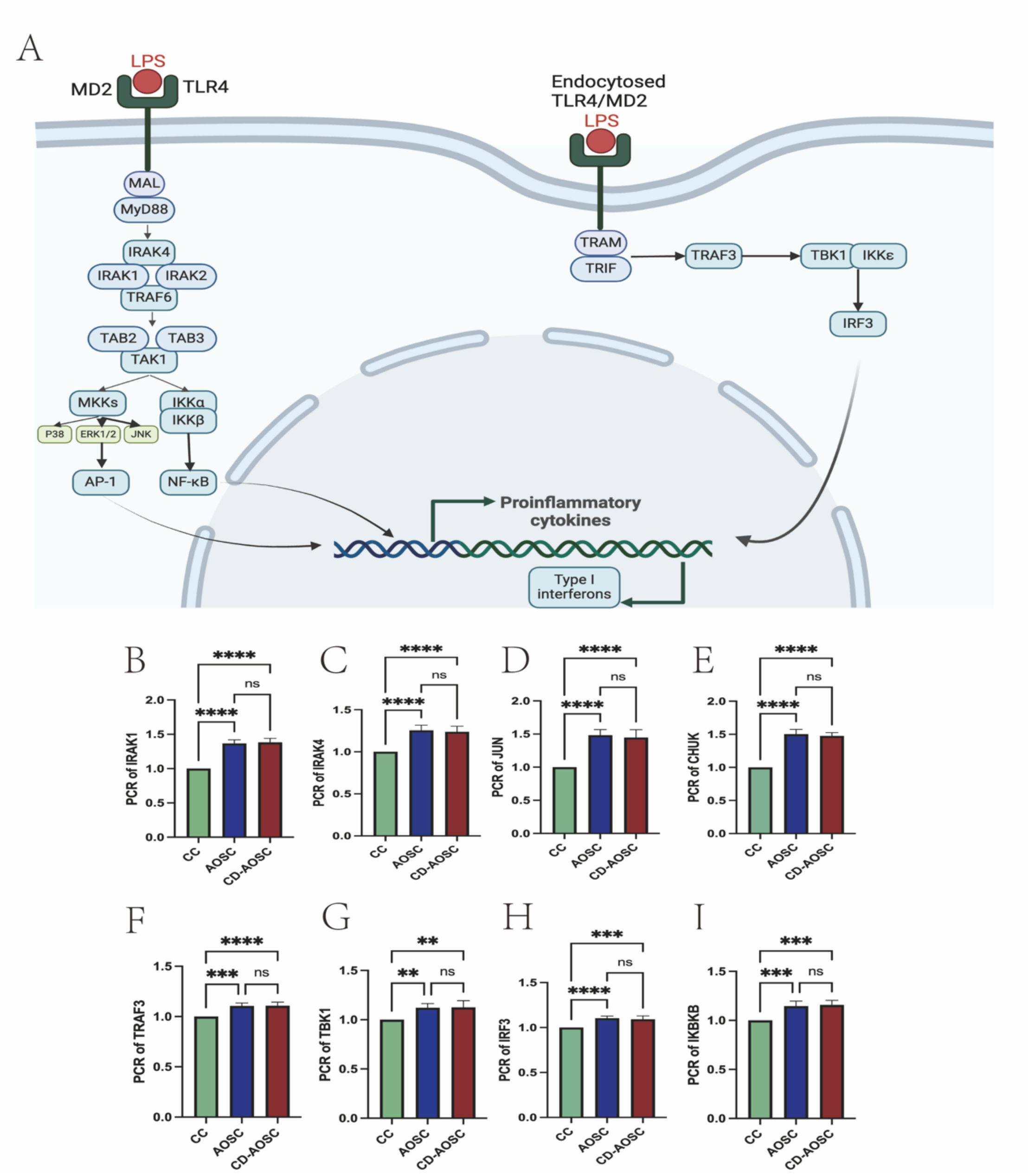

Fig.S9 Molecular testing of blood from different patient groups in the downstream of the endotoxin/TLR4 pathway.

(A) Mechanistic pathway illustrating how endotoxin (LPS) activates macrophage cells, as described in the literature(1).

(B)-(I) PCR array results for IRAK1, IRAK4, JUN, CHUK, IKBKB, TRAF3, TBK1, and IRF3 in patients' blood from the three groups. * *P*<0.05, ** *P*<0.01, * *P*<0.001, **** *P*<0.0001.**

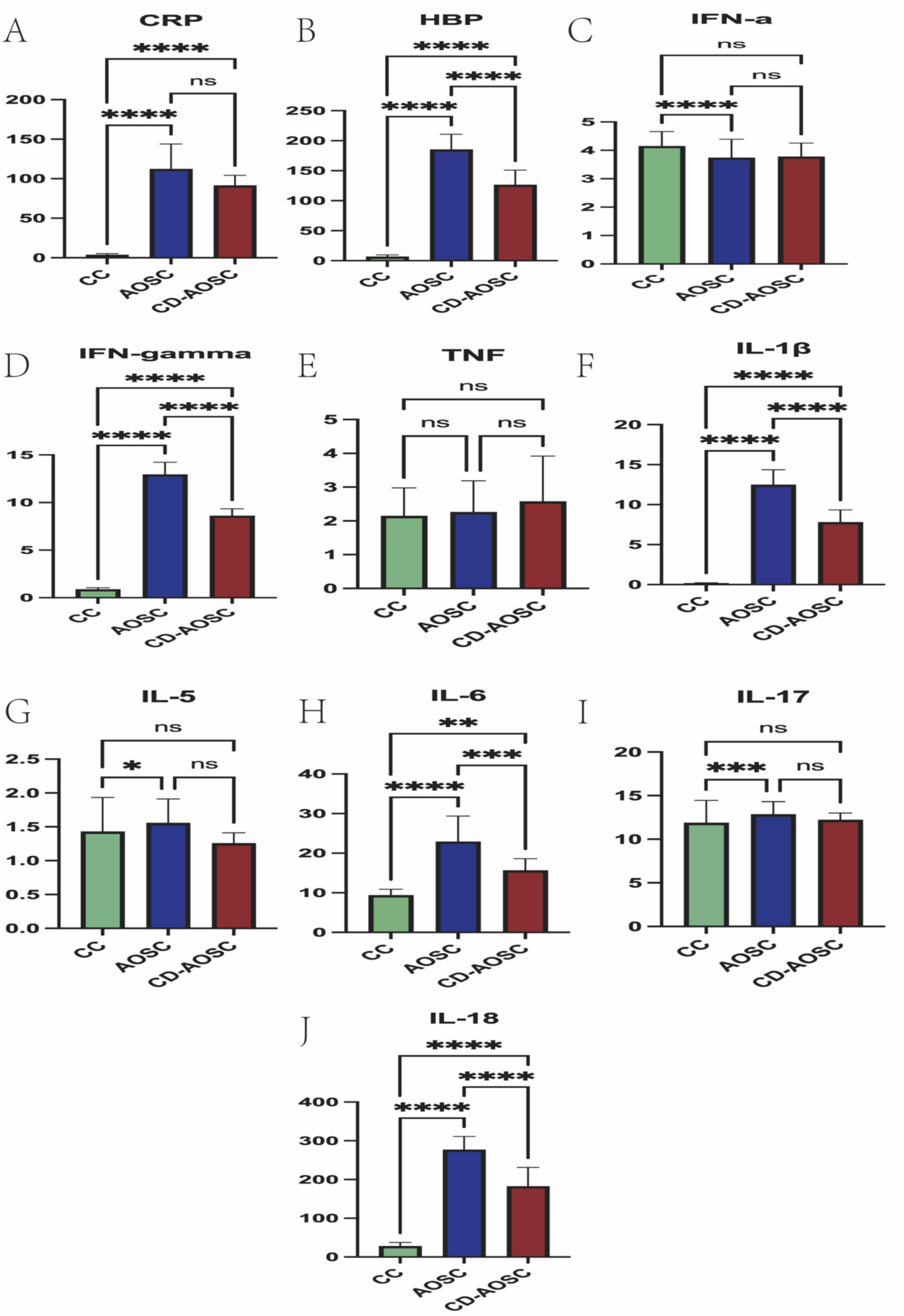

Fig.S10 Bar plot of patients' blood inflammatory markers that were significantly different as determined by ANOVA (see Table 2). * *P*<0.05, ** *P*<0.01, * *P*<0.001, **** *P*<0.0001.**

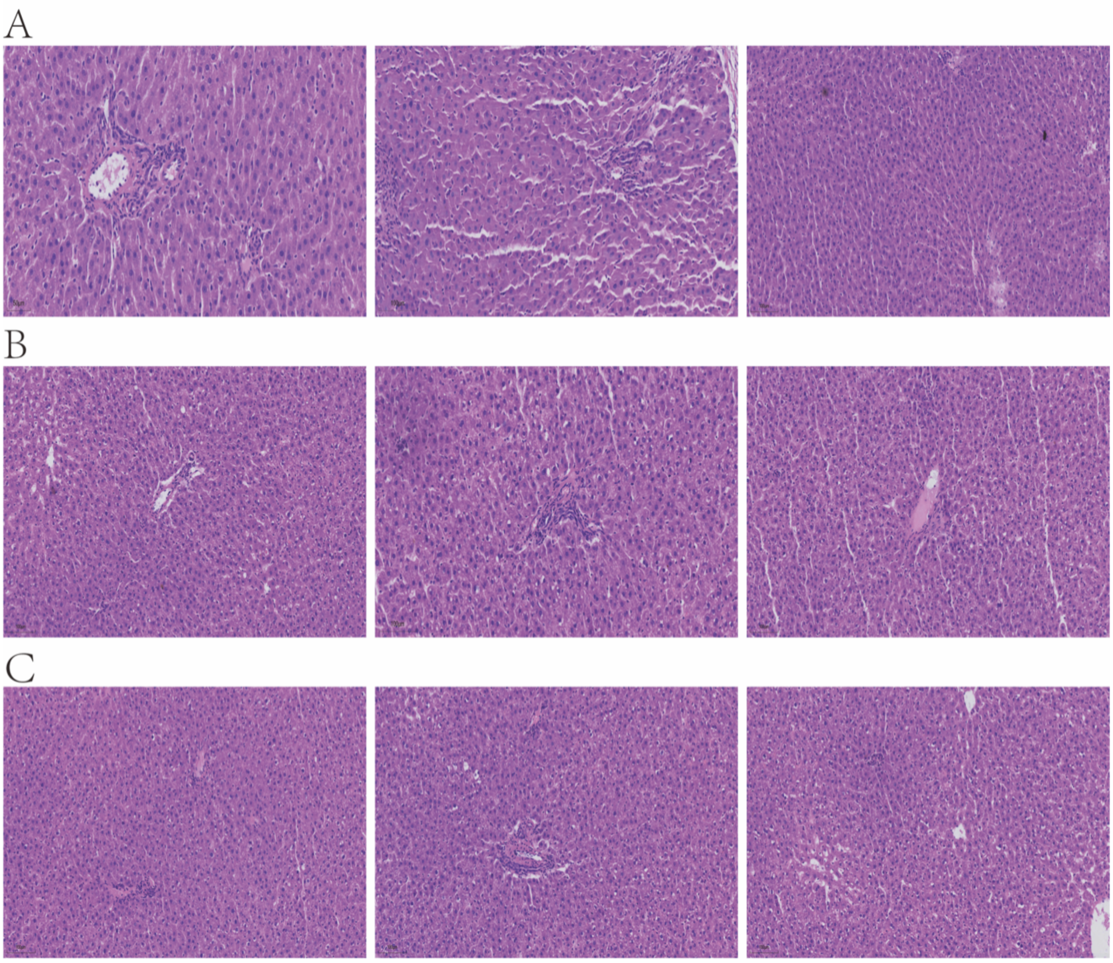

Fig.S11 Hematoxylin and eosin (H&E) stained images of liver tissues from euthanized rats seven days postoperatively in Group A. (A)-(C) represent three different slices from three different rats in Group A.

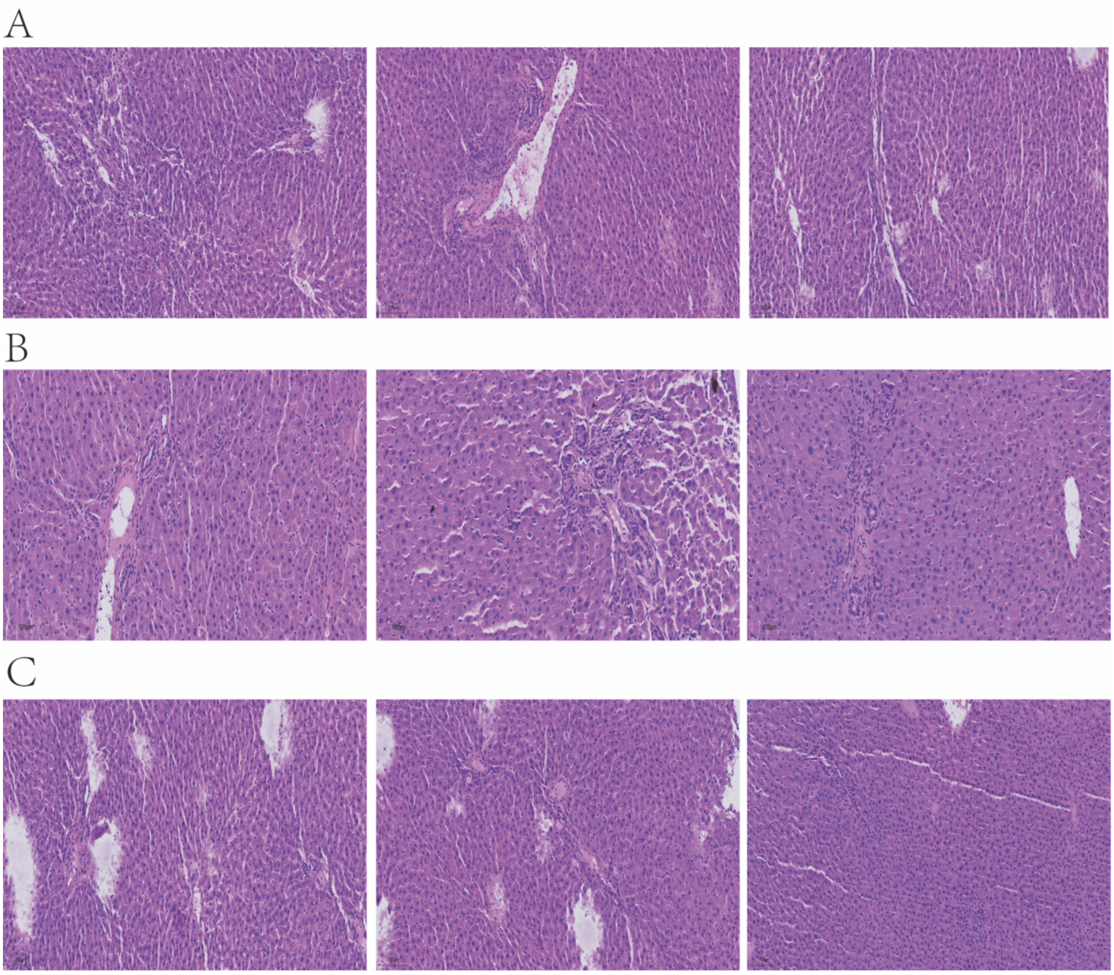

Fig.S12 Hematoxylin and eosin (H&E) stained images of liver tissues from euthanized rats seven days postoperatively in Group B. (A)-(C) represent three different slices from three different rats in Group B.

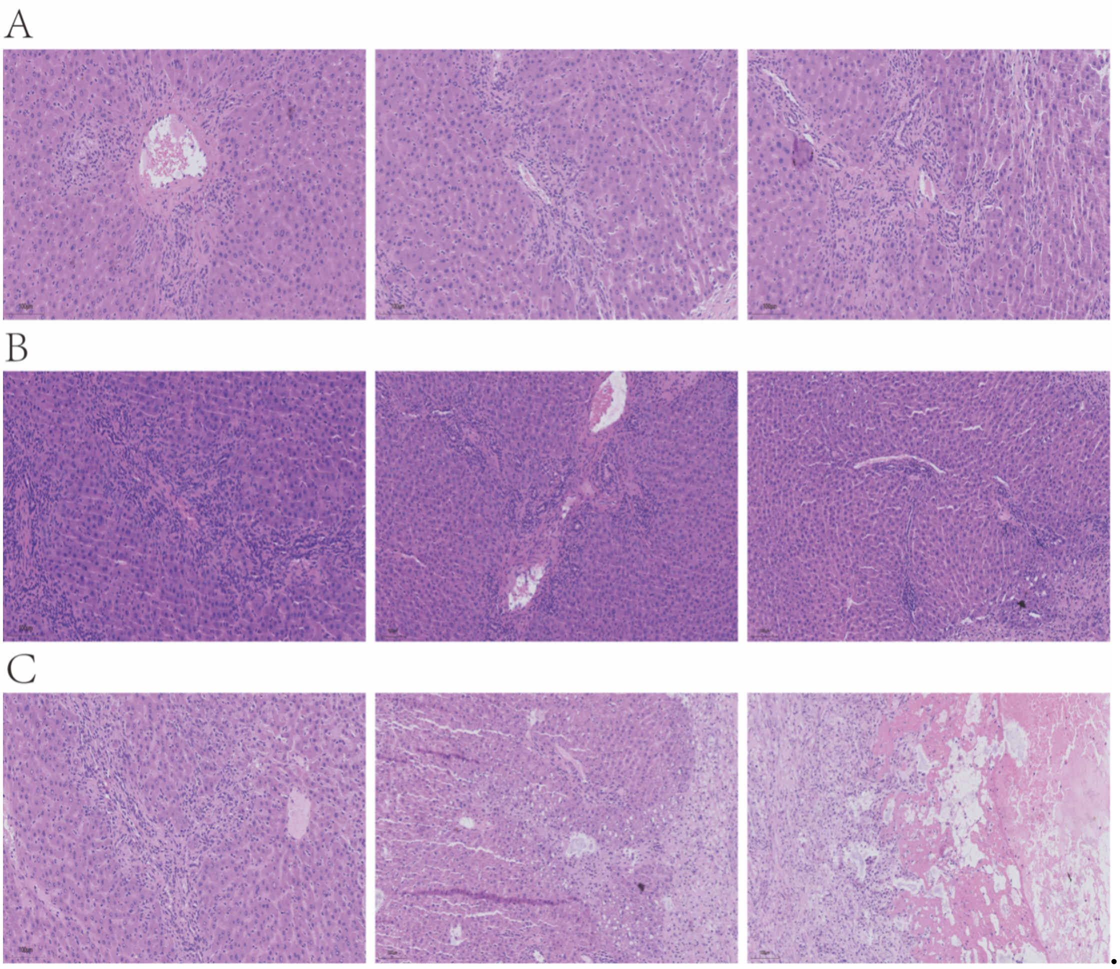

Fig.S13 Hematoxylin and eosin (H&E) stained images of liver tissues from euthanized rats seven days postoperatively in Group C. (A)-(C) represent three different slices from three different rats in Group C.

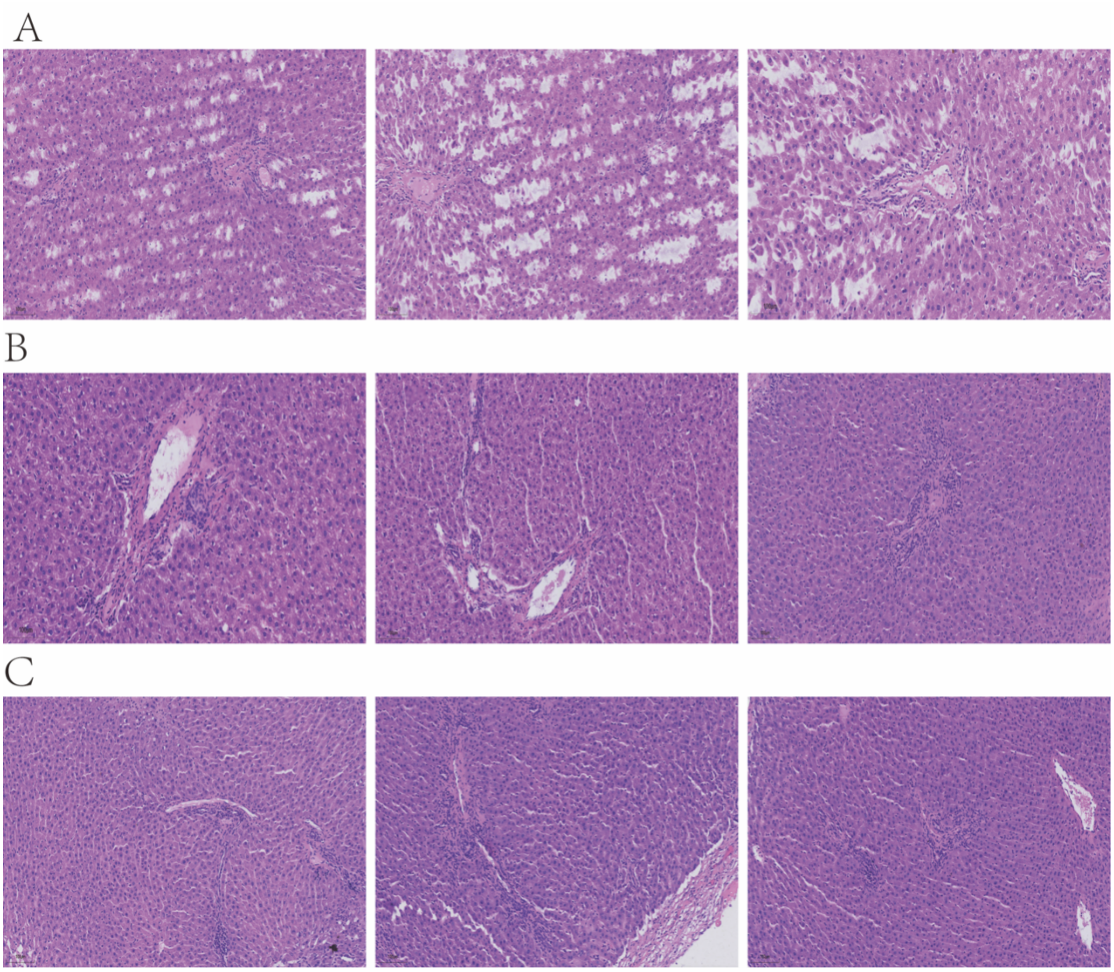

Fig.S14 Hematoxylin and eosin (H&E) stained images of liver tissues from euthanized rats seven days postoperatively in Group D. (A)-(C) represent three different slices from three different rats in Group D.

Supplementary Table

Table S1 General information statistics between Group AOSC and CD-AOSC

	AOSC (N=132)	CD-AOSC (N=5)	p value
Gallbladder gangrene			0.346
0	122 (92.4%)	4 (80.0%)	
1	10 (7.6%)	1 (20.0%)	
Surgery(0: CBDE, 1: LCBDE)			1.000
0	45 (34.1%)	2 (40.0%)	
1	87 (65.9%)	3 (60.0%)	
Time for BP Recovery	3.5 (3.0, 4.0)	33.0 (32.0, 34.0)	< 0.001
Systolic Blood Pressure on Admission	84.0 (83.0, 86.0)	85.0 (82.0, 87.0)	0.968
Diastolic Pressure on Admission	40.0 (38.0, 42.0)	47.0 (45.0, 48.0)	0.001
Operation time(min)	79.0 (71.0, 152.0)	78.0 (72.0, 149.0)	0.986

The information of group AOSC, and CD-AOSC are illustrated in design of the experiment in method part. CBDE: common bile duct exploration. LCBDE: laparoscopic common bile duct exploration. Gallbladder gangrene: 0 for no gangrene,

1 for gangrene discovered in the operation. BP: blood pressure. The statistical numbers for numeric data shown in each cell of the table was in the form of Median (Interquartile Range). The numbers for category data were summarized in counts(percentage) form. Statistical method used for comparison of continuous or category data were listed in the method part. *P* value $\leqslant$ 0.05 was statistically significant different.

Table S2 Statistics for laboratory tests between Group AOSC and CD-AOSC

	AOSC (N=132)	CD-AOSC (N=5)	p value
CRP(mg/L)	110.9 (89.4, 129.4)	91.9 (91.3, 98.4)	0.073
HBP(ng/mL)	184.5 (169.5, 201.1)	129.1 (107.5, 130.3)	< 0.001
Normetanephrine(U/mL)	133.4 (125.1, 141.1)	96.6 (94.7, 100.0)	< 0.001
Metanephrine(ng/mL)	1.2 (1.0, 1.4)	0.8 (0.7, 1.1)	0.090
Cortisol(U/L)	495.1 (393.3, 572.7)	551.5 (377.9, 587.5)	0.836
IFN-α(kU/L)	3.7 (3.4, 4.2)	3.9 (3.3, 4.0)	0.963
IFN-γ(U/L)	13.1 (12.0, 13.8)	8.7 (8.1, 9.1)	< 0.001
IL-1β(pg/mL)	12.5 (11.0, 13.8)	7.7 (6.8, 7.8)	< 0.001
IL-5(pg/mL)	1.6 (1.3, 1.8)	1.2 (1.2, 1.3)	0.031
IL-6(pg/mL)	23.2 (17.8, 28.0)	14.1 (13.8, 16.9)	0.011
IL-17(pg/mL)	13.1 (11.8, 13.8)	12.7 (11.8, 12.8)	0.213
IL-18(pg/mL)	277.9 (253.6, 301.4)	206.9 (183.6, 208.1)	< 0.001
iNOS(U/mL)	7.8 (7.8, 7.9)	3.9 (3.8, 3.9)	< 0.001

	AOSC (N=132)	CD-AOSC (N=5)	p value
PVAT Endotoxin(EU/g)	25.6 (19.4, 29.6)	233.3 (207.7, 256.1)	< 0.001
Endotoxin in Adipose(EU/g)	7.7 (5.6, 10.1)	71.0 (70.6, 72.0)	< 0.001
TBIL(μmol/L)	136.8 (109.2, 160.8)	161.5 (107.3, 171.2)	0.601
DBIL(μmol/L)	71.6 (64.9, 78.2)	87.2 (60.8, 92.2)	0.470
Hb(g/L)	131.0 (124.8, 135.0)	126.0 (126.0, 130.0)	0.667
ALT(U/L)	141.0 (126.0, 156.2)	176.0 (141.0, 178.0)	0.101
AST(U/L)	116.0 (102.0, 132.2)	110.0 (109.0, 113.0)	0.696
BUN(mmol/L)	7.2 (6.7, 7.6)	3.8 (3.1, 3.9)	< 0.001
Creatinine(μmol/L)	103.0 (95.7, 108.8)	63.5 (61.7, 64.6)	< 0.001

CRP: C-reactive protein, HBP: Heparin-binding protein, IFN: Interferon, IL: Interleukin, iNOS: inducible Nitric oxide synthase, PVAT: Perivascular adipose tissue, TBIL: Total bilirubin, DBIL: Direct bilirubin, Hb: Hemoglobin, ALT: Alanine transaminase, AST: Aspartate aminotransferase, BUN: Blood urea nitrogen. The statistical numbers for numeric data shown in each cell of the table was in the form of Median(Interquantile Range). Statistical method used for comparison of continuous or category data were listed in the method part. *P* value $\leqslant$ 0.05 was statistically significant different.

Table S3 Collinearity Assessment of Predictors in the Stepwise multivariable regression model for recovery time prediction

Parameter	VIF
IFN-γ (U/L)	71.0
IL-1β (pg/mL)	41.3
IL-18 (pg/mL)	30.7
iNOS (U/mL)	126.7
PVAT_endotoxin (EU/g)	163.1
BUN (mmol/L)	168.2
creatinine (μmol/L)	82.9

Table S4 LASSO Regression Results Identifying Robust Predictors of Time to Blood Pressure Recovery

Variable	LASSO Coefficient
Intercept	63.489
BPonadmissionsystolic	-0.037
HBP (ng/ml)	0.0002
Normetanephrine (U/ml)	-0.0023
IFN-γ (U/L)	0.061
IL-1β (pg/ml)	0.480
IL-5 (pg/ml)	0.091
IL-6 (pg/ml)	-0.008
IL-18 (pg/ml)	-0.044
iNOS (U/ml)	-6.523
PVAT_endotoxin (EU/g)	0.012

Coefficients represent the estimated effect sizes of each variable selected by the LASSO model using 10-fold cross-validation. Variables with zero coefficients (not shown) were penalized and excluded from the final model, indicating limited independent predictive value in the presence of other covariates.

Table S5 Immune cell percentage calculated by immunedeconv using transcriptome sequencing data of PVAT

	cell_type	CC1	CC2	CC3	AOSC1	AOSC2	AOSC3	CD-AOSC1	CD-AOSC2	CD-AOSC3
1	B cell	0.01981352	0.00243076	0.02294295	0.00210606	0.08608252	0.32318518	0.05385635	0.09043283	0.01976037
2	Macrophage	0.01119201	0	0.00609163	0.27633863	0.38625257	0.03556002	0.08914092	0.00324159	0.0029651
3	Macrophage	0	0.00306852	0.00234252	0.25775766	0	0.07746092	0.02328974	0.03257734	0.05511612
4	Monocyte	0.00520697	0	0.00408444	0	0	0	0	0.00630479	0
5	Neutrophil	0.01906003	0	0.01605387	0.08226853	0	0.00823908	0	0.01969367	0.27836207
6	NK cell	0.00510763	0	0.01026093	0	0.01088354	0.01703852	0.01725025	0.01257986	0.02674065
7	T cell CD4+ (	0	0	0	0	0	0.00054111	0.00978065	0.04281532	0.04925928
8	T cell CD8+	0	0	0	0.05606867	0.0146957	0.03615753	0	0.01327536	0
9	T cell regula	0.00594772	0	0	0	0.04940821	0.04653309	0.0375369	0.02004893	0.01842257
10	Myeloid der	0	0.0003758	0.01092009	0	0	0.06959407	0.01287066	0.0238601	0.03646342
11	uncharacter	0.93367213	0.99412492	0.92730357	0.32546045	0.45267747	0.38569048	0.75627454	0.73517021	0.51291042
12	immune cell	0.06632787	0.00587508	0.07269643	0.67453955	0.54732253	0.61430952	0.24372546	0.26482979	0.48708958

Table S6 GO pathway enrichment results between Group CD-AOSC and CC

Ontology	GO ID	Description	Adjust *P* value
**BP**	0002274	myeloid leukocyte activation	$1.59*10^{-26}$
**BP**	0050863	regulation of T cell activation	$1.28*10^{-12}$
**BP**	0042116	macrophage activation	$3.04*10^{-11}$
**BP**	0007249	I-kappaB kinase/NF-kappaB signaling	$3.04*10^{-11}$
**BP**	0097529	myeloid leukocyte migration	$5.43*10^{-10}$
**BP**	0051092	positive regulation of NF-kappaB transcription factor activity	$4.14*10^{-9}$
**BP**	0043123	positive regulation of I-kappaB kinase/NF-kappaB signaling	$1.05*10^{-7}$
**BP**	1904407	positive regulation of nitric oxide metabolic process	$4.06*10^{-5}$
